# Measuring and modelling the structural dynamics of poricidal anthers: a mechatronic approach

**DOI:** 10.1098/rsif.2025.1321

**Published:** 2026-09-09

**Authors:** Siyang Zhou, Georg Schitter, Ernst Csencsics

**Affiliations:** Institute of Mechatronics and Power Electronics, Vienna University of Technology, Vienna, Austria

**Keywords:** buzz pollination, anther dynamics, vibration measurement, finite-element modelling, multi-body dynamics, Melastomataceae, plant biomechanics

## Abstract

The mechanical dynamics of poricidal stamens play a crucial role in buzz pollination, but they have only been studied in a few species showing comparatively less curvature. This paper presents an integrated experimental–computational approach to characterize the dynamic behaviour of the morphologically more complex stamens of *Medinilla magnifica*. An experimental set-up employing a custom-built shaker and a laser triangulation sensor is developed to measure the stamen motion with high spatial and temporal resolution. The resulting displacement transmissibility reveals three distinct resonances, with the second one falling within the reported frequency range of pollination buzzes. Finite-element and multi-body models are developed and validated against experimental results. The models further reveal how the stamen morphology gives rise to its complex dynamic behaviour. The developed method enables precise parametric modelling of stamens and provides new insight into the mechanical basis of pollen release, thereby offering a framework for studying buzz pollination dynamics across plant taxa.

## 1. Introduction

Buzz pollination is a pollination strategyin which bees actively release pollen by vibrating flowers with poricidal anthers at 100–400 Hz [1]. Unlike most flowers presenting their pollen on exposed anther surfaces through longitudinal dehiscence, poricidal anthers restrict pollen release to small terminal pores or slits. This morphology is found in over 20 000 plant species across at least 65 families, including many ecologically and economically significant taxa [1–3]. Successful pollination of these plants depends on the ability of bees to generate vibrations with their indirect flight muscles, a behaviour absent in honeybees but widespread among bumblebees and numerous solitary bee lineages [4,5].

The evolutionary and ecological implications of buzz pollination are profound. Poricidal anthers may act as filters, limiting access to pollen rewards to only those visitors capable of producing the appropriate vibrations [6,7]. Such specialization has consequences for pollinator community composition, plant reproductive success and ultimately for the diversification of floral morphologies [4,8]. At the same time, buzz pollination is crucial for the productivity of crops such as tomato, aubergine and blueberry, where efficient pollen release cannot be achieved without vibratory stimulation [9,10].

Despite this rich body of ecological and evolutionary work, the physical mechanism of pollen release remains poorly understood. Pollen release, as a biological interaction between bees and plants, is a biomechanical process in which floral structures transmit and transform vibratory energy into the periodic motion of anthers [11,12]. The geometry, stiffness and mechanical properties of poricidal anthers directly influence how anthers respond to the pollination buzz of bees and how efficiently pollen is expelled [3,4]. Earlier work by King & Lengoc [13] established foundational methods for measuring vibrational pollen collection dynamics, providing a basis for subsequent structural dynamics studies.

In recent years, many new studies have focused on the response of poricidal anthers to dynamic excitations. These studies often anchor a stamen on a shaker oscillating at varying frequencies and measure the response at the anther tip with a laser Doppler vibrometer. Nunes *et al.*[14] found that ‘the average natural frequency of stamens across six *Solanum* taxa varied from 44.57 ± 1.36 Hz (mean ± SE) for pollinating stamens of *S. citrullifolium* to 294.30 ± 47.37 Hz for the feeding stamens of *S. grayi var. grayi*’. Jankauski *et al.* [15] reported that the first and second resonances of *Solanum elaeagnifolium* anthers are around 64 and 1228 Hz. Furthermore, they measured the response at multiple locations along the anther to describe the deflection shape and compared this with a finite-element (FE) model of the anthers. Xu *et al.* [16] further extended the biomechanical perspective to Pedicularis (Primulaceae), demonstrating size-dependent associations between bumblebees and flowers with a morphology distinct from that of *Solanum*. More recently, Shi *et al.* [17] proposed a discrete element simulation of pollen grain motion within tomato anthers, complementing structural approaches by modelling pollen ejection at the particle level.

**Figure 1.**
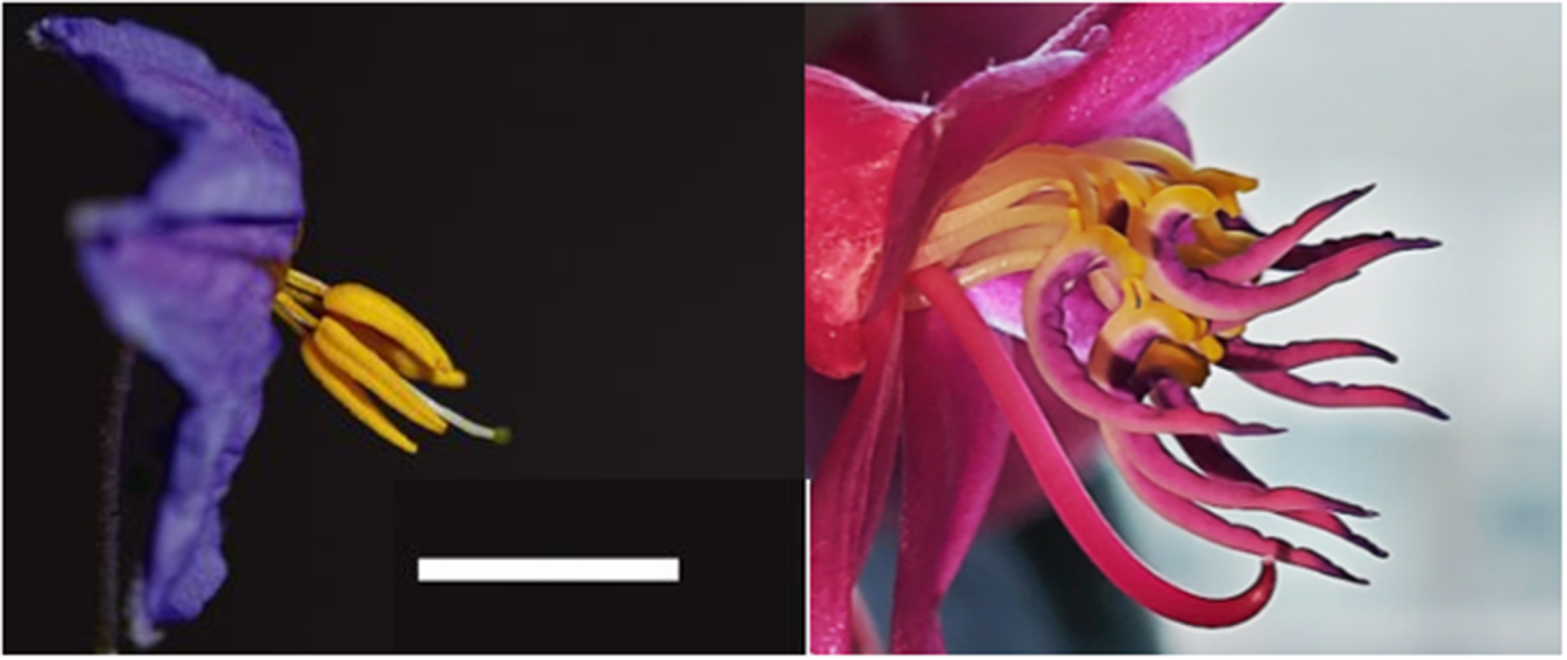
Comparison of the geometry of the stamens of *S. elaeagnifolium*, scale bar is 10 mm, modified from Vallejo-Marín *et al.* [7] (CC BY-NC) (left) and *M. magnifica* (right). *Solanum* usually have stiffer and straighter anthers and an apical pore, and the anther is much longer compared to the filament, whereas stamens of Melastomataceae commonly have long filaments, connective tissues with complex appendages or elongations and markedly curved anthers with corrugated thecal walls. Scales of *M. magnifica* stamen are presented in figure 6a.

Building on these results, the next step is to understand how different stamen morphologies influence vibration transmission and pollen release. Previous structural dynamics studies have established that *Solanum* stamens behave largely as rigid bodies pivoting at the filament base, with natural frequencies that fall outside the buzz pollination range unless bee mass is considered [15]. However, it remains unclear whether this picture extends to the morphologically diverse stamens found across the many families of buzz-pollinated plants. Most previous work has focused on *Solanum*, a genus with relatively straight and rigid anthers. To broaden the biomechanical perspective to morphologically more curved stamen types common among other buzz-pollinated lineages, such as the large plant family Melastomataceae, we examine the stamens of *Medinilla magnifica* (figure 1), a taxon with a markedly different stamen morphology. Its pronounced curvature and composite anther–filament structure provide an opportunity to study the dynamic response of non-*Solanum* anther morphology.

Furthermore, while previous studies mainly focused on amplitude spectra of the stamen's response to vibrational excitation, a comprehensive mechanical analysis also requires the phase component of the dynamic response and its spatial variation along the stamen. Such information reveals how vibrational energy propagates through the structure, whether different regions move independently, and enables direct validation of numerical models [18]. By combining the detailed dynamic response with numerical modelling, the mechanical understanding of buzz-pollinated stamens can be advanced beyond resonance identification, towards a full description of their dynamic behaviour.

The contribution of this study is a method to measure and model the structural dynamic response of poricidal anthers. The stamens of *M. magnifica* are used as the focal system to extend the biomechanical perspective beyond *Solanum*. The family Melastomataceae exhibits exceptional stamen disparity, including widespread heteranthery, elaborate connective appendages and repeated evolutionary shifts in stamen morphology, making it an ideal model for investigating how structural dynamics evolve with function in buzz-pollinated systems [19–22] and opening our perspective to the floral biomechanics of morphologically complex stamens. The proposed method captures dynamic responses at multiple locations along the stamen, providing a complete description of its dynamic behaviour. To interpret the measured dynamic responses mechanistically, a high-fidelity FE model and a simplified multi-body model are developed and validated to complement the experiments. These two computational modelling approaches together allow us to link measured motions to underlying deformation modes and to establish a framework for future analyses of how factors such as anther morphology, bee mass and bite location (where bees apply pollination buzzes) influence vibrational response during pollination.

The remainder of this paper is organized as follows. The Methods section describes the experimental set-up and modelling procedures. The Results section presents the measured dynamic response of *M. magnifica* stamens, followed by the FE analysis (FEA) and multi-body model and their comparison with experimental results. The Discussion includes the implications of these findings for the study of buzz-pollinated anthers. Additionally, supplementary analyses presented in the appendices demonstrate the generalizability and capability of the multi-body model through a subregional parameter study, a stiffness identifiability analysis and a cross-specimen geometry study.

## 2. Methods

### 2.1. Description of stamen dynamic behaviour

**Figure 2.**
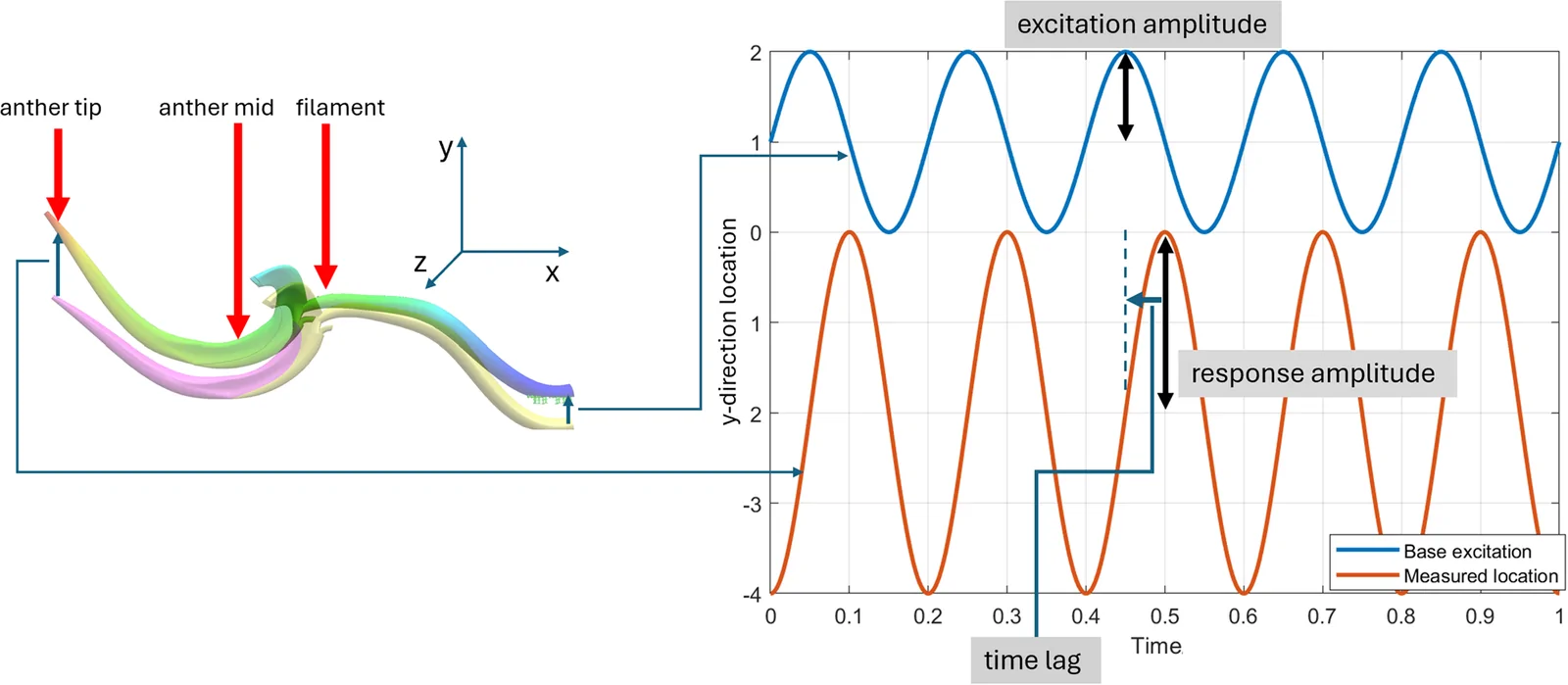
Illustration of displacement transmissibility, measurement locations (red arrows) and definition of coordinates. The stamen model (left) is excited sinusoidally at its filament base, and the response motion at marked locations is measured. The plot (right) shows an example of the base excitation and the response at a single frequency at the anther tip.

The dynamic behaviour of the stamen is described using its displacement transmissibility, which quantifies how motion at a given point along the stamen responds to vibrational motion imposed at its base. Giving a base vibration to the stamen is a standard structural dynamics measurement technique, because it is simple, controllable and less likely to suppress dynamic features that direct excitation at a single point, mimicking the bee buzz, for example, might obscure. When the base is excited with a small sinusoidal motion, its response motion can be assumed linear, and the response motion at any measured location can be approximated by a sinusoidal signal of the same frequency but with a different amplitude and phase, as illustrated in figure 2. In this representation, the ratio of response amplitude to excitation amplitude defines the amplitude of the transmissibility, while the time lag between the two signals, expressed as a fraction of the vibration period, defines its phase component. Together, these two quantities describe how strongly and how promptly the structure responds to excitation at a given frequency. By repeating this excitation–response measurement across a range of frequencies, a complete dynamic response can be obtained, in which the amplitude and phase of the transmissibility are shown as functions of frequency. Peaks in the amplitude curve correspond to resonances of the stamen, while the phase shifts reveal how energy is stored and dissipated within the structure.

To fully characterize the stamen's dynamic behaviour, transmissibility is measured at three representative locations along its length: the filament, the anther mid and the anther tip. These positions correspond to regions that may differ in structural function and mass–stiffness distribution. Measuring at multiple locations allows the stamen's deformation pattern to be reconstructed, rather than inferred from a single measurement. In addition, comparing transmissibility among these locations provides a direct basis for validating numerical models, since an accurate model must reproduce not only the resonance frequencies but also the relative motion patterns along the entire structure.

### 2.2. Selection of instruments

The experimental set-up requires two main components: an actuator to excite the stamen and a sensing system to measure its response. In previous studies, a common choice of actuator has been a commercially available shaker [14,15], which provides stable output but lacks clear precision on output displacement and flexibility in how the stamen can be attached. To overcome this limitation and to obtain a deterministic dynamic behaviour of the shaker, a custom-built shaker driven by a voice coil actuator (Sup Motion, Suzhou, People's Republic of China) is adopted for the present set-up. Details of the shaker design are provided in §2.4. The voice coil is powered by a custom-made amplifier.

The sensor must offer sufficient resolution and frame rate to capture the stamen motion required for computing the dynamic response. In addition, it should have a measurement range large enough to accommodate the full amplitude of the stamen's response and should be minimally influenced by z-direction movement (the coordinate system is defined in figure 2). Considering these requirements, a laser-line triangulation sensor [23] is selected. A laser triangulation sensor achieves high accuracy in distance measurement by reading the location of the image of the laser spot on the subject surface. When the laser array is aligned to the z-direction of the stamen, the disturbance of the z-direction movement can be spatially averaged out. The chosen model, Micro-Epsilon scanCONTROL 2950-10/BL (Micro-Epsilon Messtechnik GmbH, Ortenburg, Germany), has a measurement linearity of 1 µm. The measurement field has a depth of 1.2 mm and a width of 1 mm at a measurement rate of 2000 Hz, which is sufficient for measuring the dynamic response up to 800 Hz. The blue laser also works better on the slightly translucent stamen surface than the commonly used red laser. Manual stages are used to position the sensor and translate its measurement field along the sample.

### 2.3. Structure of experimental set-up

**Figure 3.**
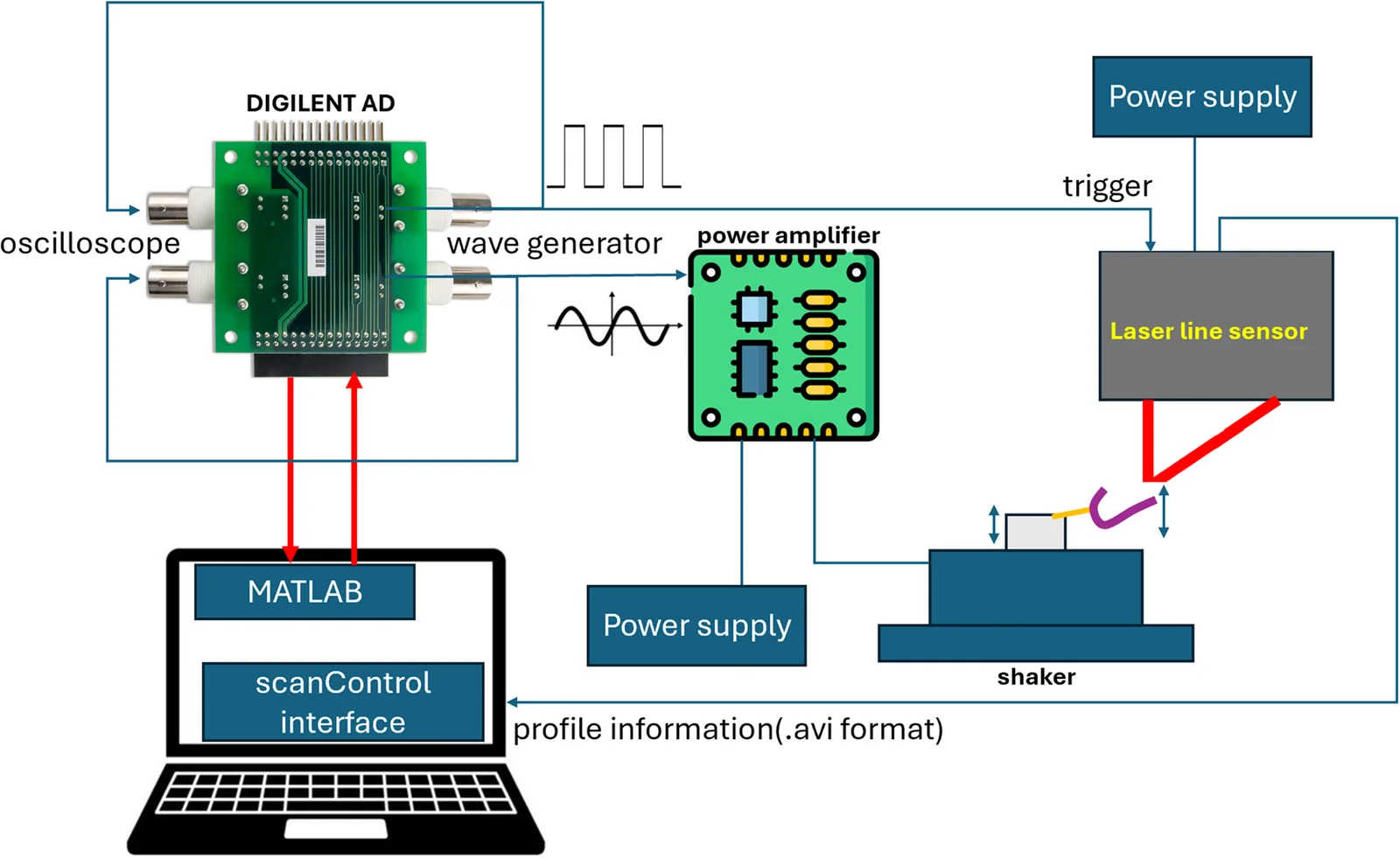
Structure of the experimental set-up. The MATLAB (The MathWorks, Inc., Natick, USA) toolbox of Digilent controls the Digilent Analog Discovery 3 to send reference signals to the power amplifier and trigger signals to the laser-line sensor via its two wave generator channels, and to record these signals with the oscilloscope channels. The power amplifier sends a sinusoidal current to the shaker to generate sinusoidal motions. When triggered, the laser-line sensor measures the displacement of the stamen and sends the data to the scanCONTROL interface software on the computer.

Figure 3 illustrates the structure of the experimental set-up. A Digilent Analog Discovery 3 (Digilent Co., NI, Austin, USA) paired with a Digilent AD BNC converter is used to generate the reference signal for the power amplifier and the trigger signal for the sensor. As the two wave generator channels are not synchronized, both signals are simultaneously recorded by the two oscilloscope channels to ensure proper synchronization. The sensor output is transmitted to the scanCONTROL interface software (scanCONTROL Configuration Tools, Micro-Epsilon Messtechnik GmbH, Ortenburg, Germany) and stored as AVI files for subsequent processing.

The reference signal sent to the power amplifier is a sinusoidal voltage with a defined frequency and amplitude, while the trigger signal to the sensor is a square wave. The amplitude of the square wave follows the sensor specifications, and the duration of its ‘on’ state varies with the excitation frequency to ensure that appropriate motion periods are recorded. Typically, the reference signal runs for more than 60 cycles so that there is enough time for the stamen to build up a steady response. The sensor then records the stamen displacement for at least 20 cycles, providing a measurement duration sufficient to reduce noise via averaging.

### 2.4. Design of the stamen shaker

**Figure 4.**
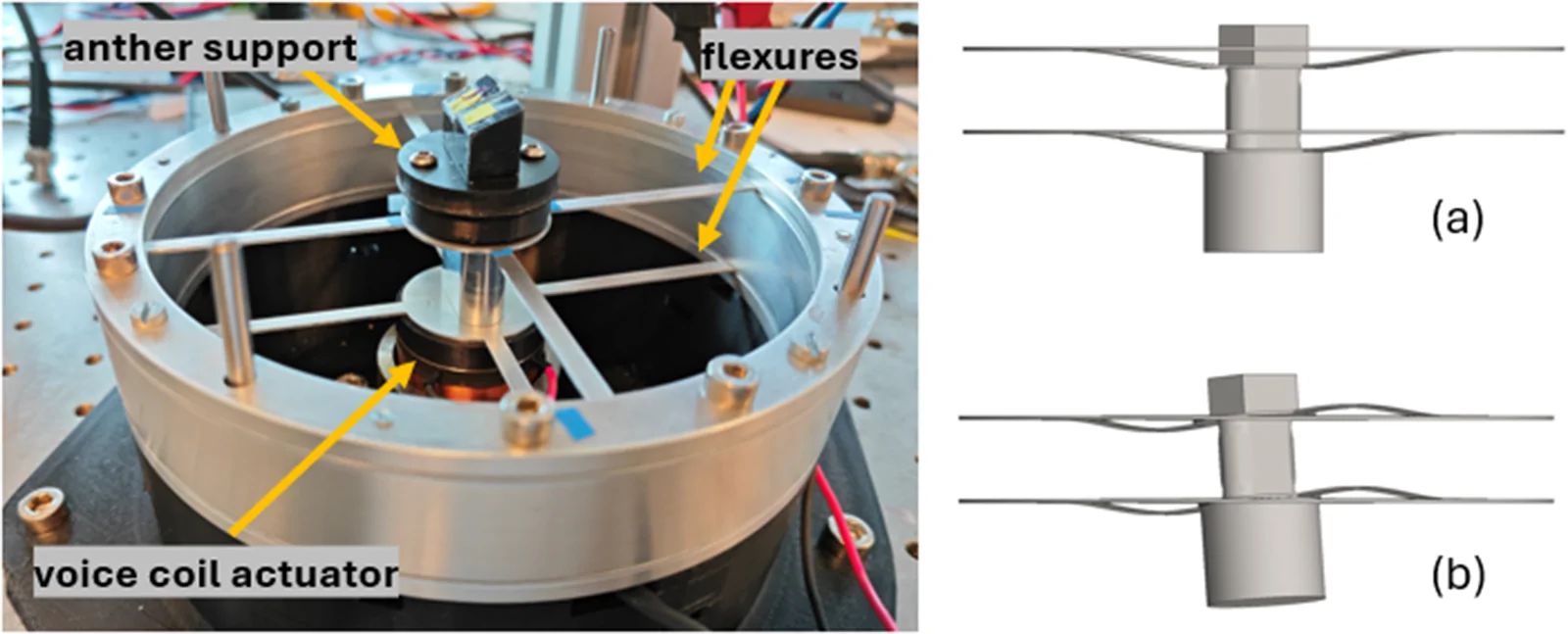
The designed shaker (left). It consists of a three-dimensional printed base housing that can be mounted on an optical table. Two flexures are coupled by a ring spacer and a central spacer, which are fastened together with screws and fixed to the base housing. A stamen support is attached on top of the flexures. The magnet of the Lorentz actuator is fixed to the housing, while the coil is fixed to the flexure assembly. Mode analysis of the FEA (right), (a) the suspension mode of the shaker is at 57 Hz and (b) the tip-tilt mode of the shaker is at 1080 Hz.

The ideal excitation motion applied to the sample should involve only one degree of freedom in the vertical direction. According to the screw theory of flexure [24], a single thin flexure element simultaneously transmits both the translational motion and an undesired tip-tilt motion. To suppress the latter, two identical flexures are arranged in parallel with an offset between them (figure 4, left), so that the coupled tip-tilt motion is effectively cancelled while preserving the pure vertical translational motion.

Because the geometry of the flexures and their offset determine how effectively the tip-tilt motion is suppressed, a FEA is performed to predict the system's dynamic characteristics. As shown in figure 4a,b (right), the fundamental suspension mode occurs at 57 Hz, whereas the tip-tilt mode appears at 1080 Hz. This result indicates that, within the frequency range of interest (50–800 Hz), the contribution of tip-tilt motion is negligible.

**Figure 5.**
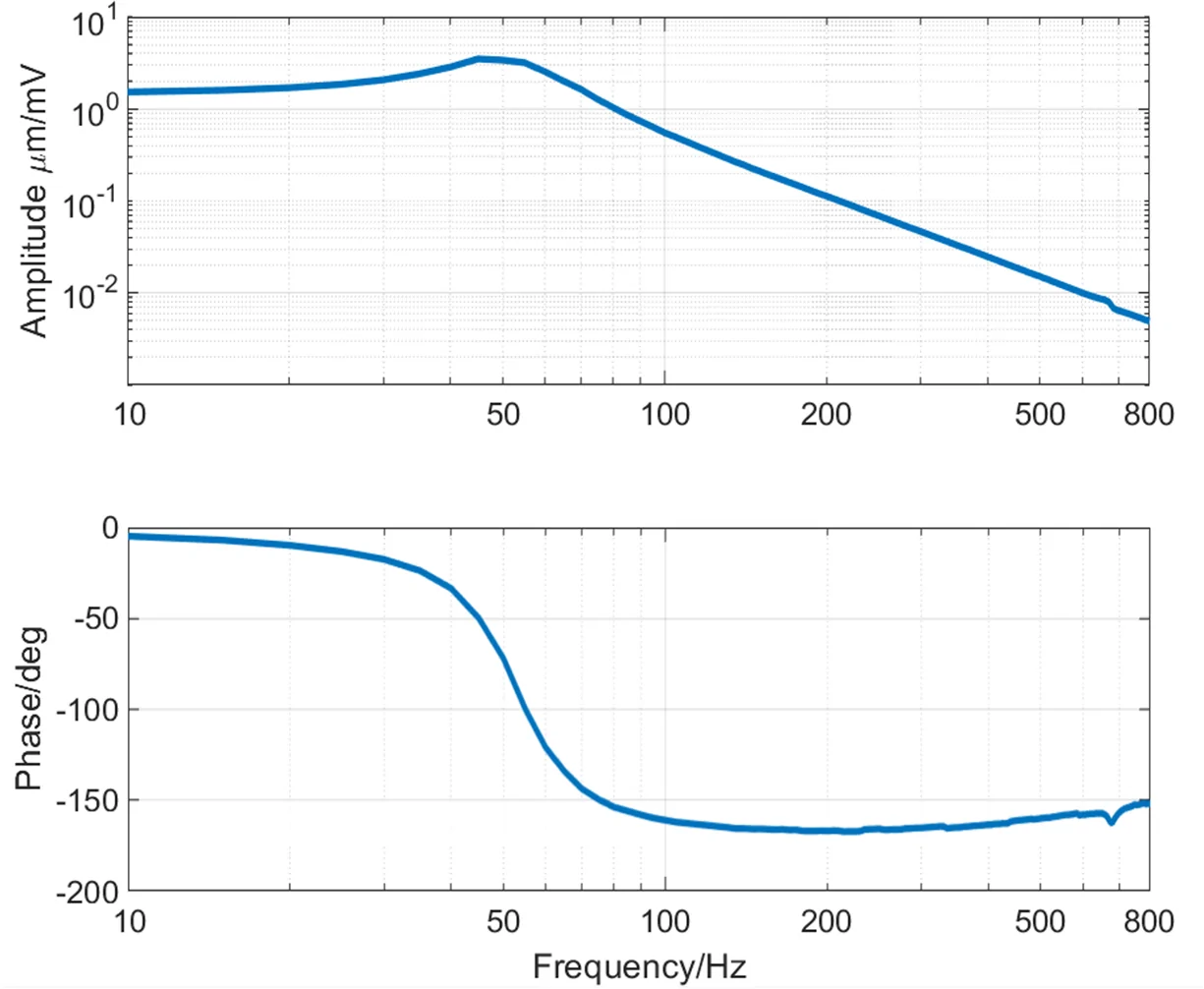
The voltage-to-displacement response of the shaker from 10 to 800 Hz. At low frequencies, the amplitude is constant and the phase is $0^{\circ }$. The suspension mode occurs at around 50 Hz, close to the FEA prediction. Beyond the resonance, the amplitude decreases by a factor of 100 for every tenfold increase in frequency, and the phase exhibits a $180^{\circ }$ phase lag.

After the system is manufactured and assembled, the reference voltage-to-displacement response of the shaker is characterized using the experimental set-up described above, with the laser sensor measuring the motion of the stamen support (data processing described in §2.6). Figure 5 shows the relation between the amplitude of the reference signal sent to the power amplifier and the resulting displacement amplitude of the stamen support at each frequency. This is the characteristic behaviour of a second-order mass–spring system, in which the moving mass corresponds to the voice coil, and the restoring stiffness is provided by the flexures. In the MATLAB script controlling the wave generator of the Digilent Analog Discovery 3, this frequency response is inverted and implemented as a feed-forward controller, allowing precise control of the displacement amplitude of the stamen support over the entire experimental frequency range.

**Figure 6.**
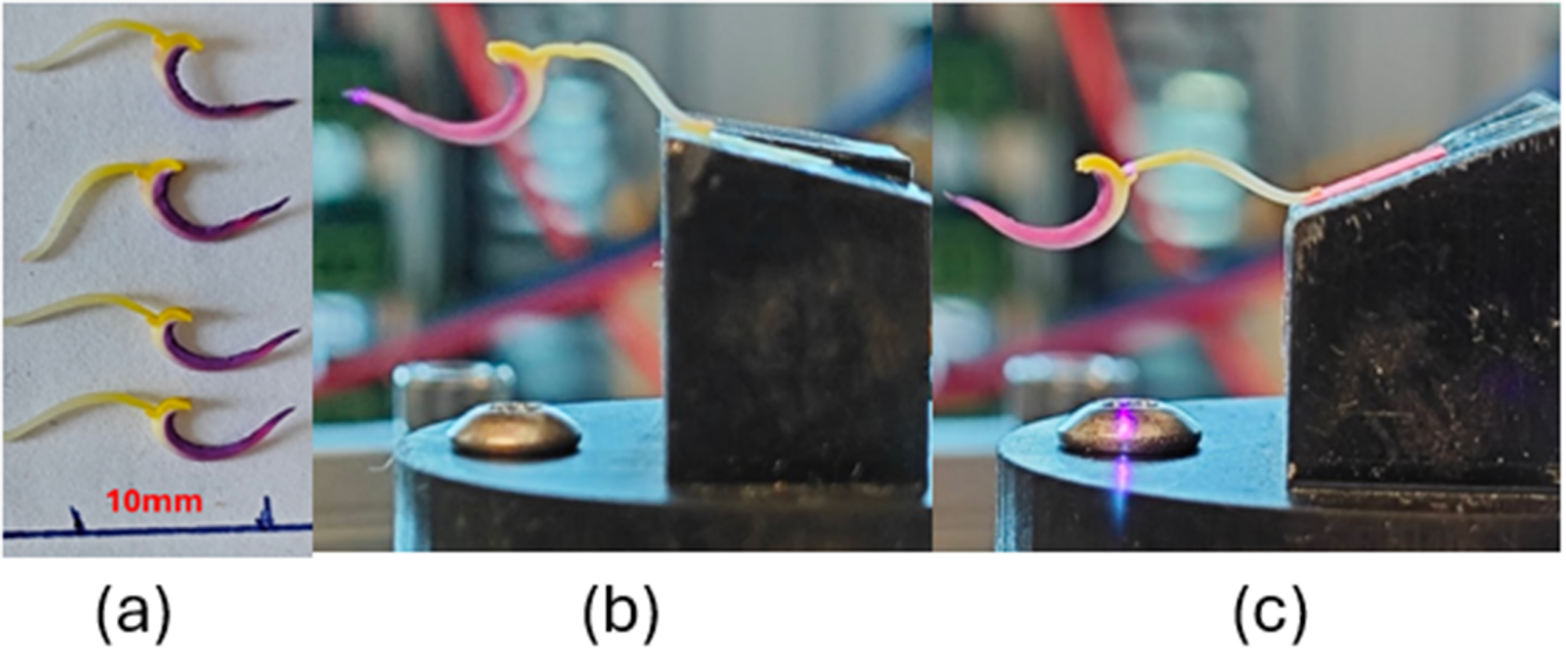
Different curvatures of stamens and the correction of stamen supports. (a) Filaments of *M. magnifica* stamens have different curvatures and need to be compensated by the inclination of stamen supports. (b,c) How two stamen supports with different inclinations keep the anthers of two stamens with different filament curvatures in a similar orientation (the orientation of the filament–anther junction–anther tip line).

The stamen is attached to the shaker using fast-drying glue. Clamping was tested in preliminary experiments but was found to accelerate water loss in the stamens, as it can easily cause damage to the clamped area. The stamen supports are stereolithography-printed blocks with different inclination angles and are connected to the shaker using two screws. These inclinations help to adjust the orientation of the stamens so that the anther is always horizontally aligned in a comparable position, as shown in figure 6.

### 2.5. Experimental procedure

Plant materials were obtained from the Core Facility Botanical Garden at the University of Vienna. Stamens were collected from two individual plants, each having multiple open flowers everyday, and at most, two stamens were taken per flower. All experiments are conducted in the morning, no later than 13.00, using flowers that are freshly opened on the same day. A single flower is cut from the plant with a sharp pair of scissors and brought to the laboratory, with its stem wrapped in wet paper tissue to preserve freshness. Before each measurement, a stamen is carefully removed from the flower by cutting at the very base of the filament to keep it intact as long as possible, since trimming would shorten the structure and alter its dynamic behaviour. After removal, the stamen is transported in a folded piece of paper to prevent tissue damage. The bottom of the filament is first dipped in fast-drying glue to seal the cut surface and provide an adhesive layer, and then attached to a small piece of paper that has been glued onto the stamen support, which gives faster and firmer adhesion.

Before each measurement, the sensor's measurement field is adjusted with manual stages and the interface software to ensure that the stamen motion is fully captured throughout the test. Once alignment is confirmed, the excitation and trigger signals are sent to the shaker and sensor, respectively. In each frequency, the amplitude of the excitation signal is back-calculated from the relation in figure 5 to generate a sinusoidal motion with an amplitude of 6 µm in the shaker. Three locations, the anther tip, anther mid and filament, as shown in figure 2, are measured sequentially to obtain a comprehensive characterization of the stamen's dynamic response. Measurements at the anther tip are typically started within 5 minutes after stamen removal, followed by the anther mid measurement about 2 minutes later and the filament measurement about 2 minutes after that of the anther mid.

To examine the effect of drying time on the dynamic response, a second round of measurements at all three locations is repeated approximately 7–8 minutes after the first set, and a third round is performed around 13 minutes after the initial measurement. This sequence is repeated until visible deformation of the stamen occurs owing to drying.

### 2.6. Data processing

The files recorded by the sensor are exported as tabular data, with each file containing the position information of one frame (i.e. one exposure). For each frame, the average y-coordinate of all points is taken as the vertical position of the measured location. Points near the edge of the stamen cross-section often exhibit larger random variations than those in the flatter central region. To improve the signal-to-noise ratio, points from the steeper edge regions are therefore omitted for averaging.

After obtaining the average vertical position in all frames of a measurement, the resulting time series is processed using a discrete Fourier transform, yielding the voltage-to-displacement response of the combined shaker–stamen system. By subtracting the previously measured voltage-to-displacement response of the shaker, the displacement transmissibility from the filament base to the measured location is obtained. The displacement transmissibility determined at the three measurement locations along the stamen is then used to characterize its structural dynamics.

Because measurements are performed sequentially at three locations along the same stamen, gradual moisture loss during the process leads to a decrease in stiffness over time, which in turn causes a slight downward shift in the resonance frequencies at the anther mid and the filament. As a result, the measured transmissibility curves from different locations could exhibit small frequency offsets even though they reflected the same underlying structural modes.

To compensate for this effect and to enable a consistent comparison among locations within a single sample, the first and second resonances at the anther mid and the filament are mapped to those at the anther tip using first-order scaling. Furthermore, the transmissibility data are interpolated to improve the frequency resolution from 5 to 1 Hz, facilitating subsequent analyses based on the experimental results.

### 2.7. Correction of measurement artefact

**Figure 7.**
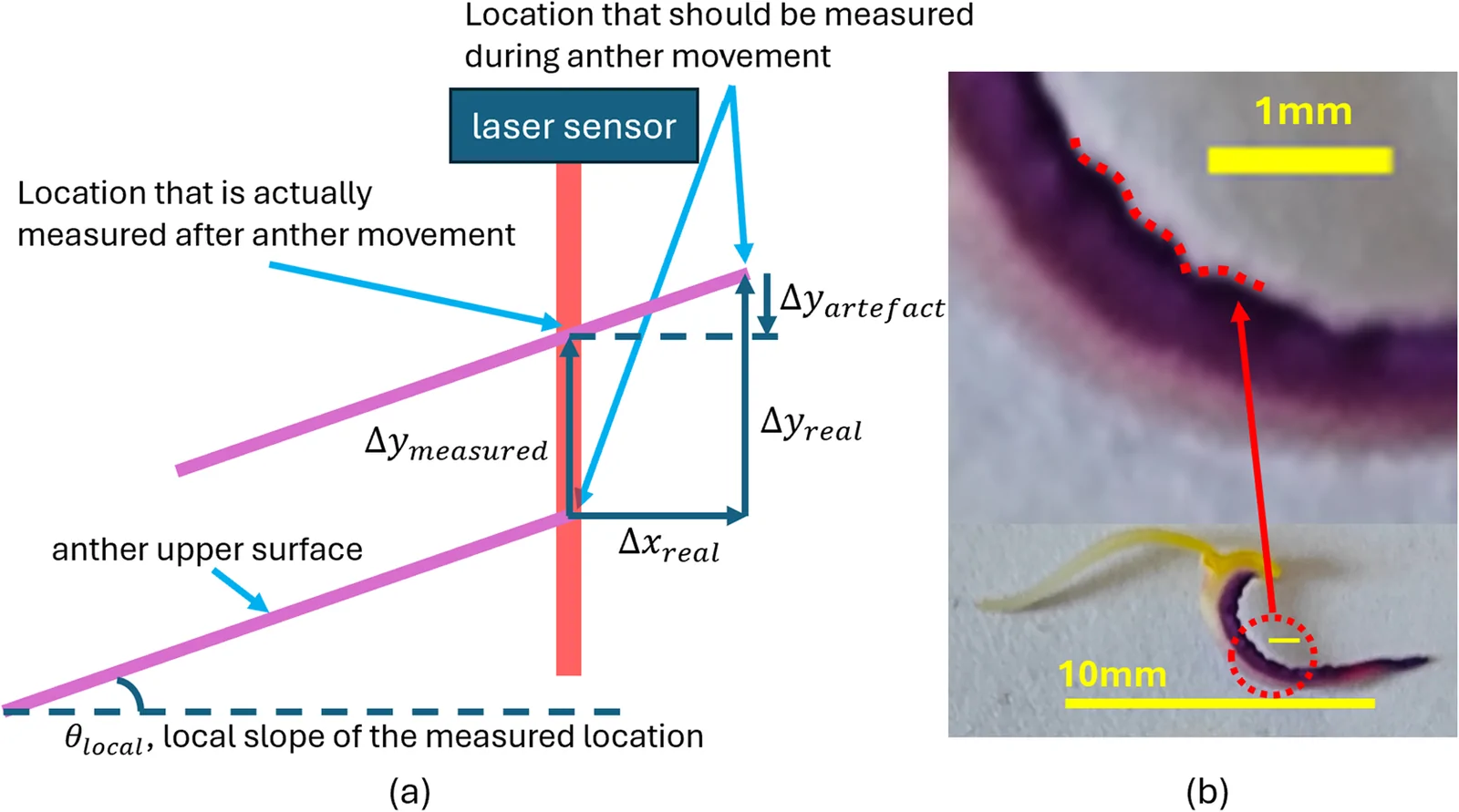
Mechanism of the measurement artefact. (a) Schematic illustration of the measurement artefact caused by the anther moving also in the x-direction and the laser failing to track the same point. (b) An example of microscopic surface texture, the corrugated thecal wall, is marked by the dotted curve.

It is important to note that, in the stamen measurements, the sensor read-out does not reflect the motion of a physical point on the stamen. As shown in figure 7a, each point on the stamen moves not only in the y-direction but also in the x-direction, with the y-motion being dominant. The laser spot does not continuously track a single physical point but shifts slightly around the target area, partly leading to a false deflection. The calculated transmissibility derived from the sensor read-out contains a measurement artefact, which can be described by

$$\Delta y_{\text{artefact}}=\Delta x_{\text{real}}\cdot \tan\theta_{\text{local}}.$$

This measurement artefact cannot be directly corrected from the experimental data, as the stamen's geometry prevents the measurement of its x-direction motion using the laser sensor. However, since both the x- and y-direction motion are available in the numerical models (described in §2.8), the transmissibility, including the measurement artefact, can be synthesized numerically and compared with the experimental results.

It should also be emphasized that the local surface slope at the measured spot arises not from the overall shape of the stamen but from its microscopic surface texture (as shown in figure 7b), given that the vibration amplitude in the experiments is on the micrometre scale. Therefore, when synthesizing the artefact, the value of the local slope cannot be taken from a specific stamen sample but is a tuning variable in the post-processing of the modelling results.

### 2.8. Finite-element modelling

**Figure 8.**
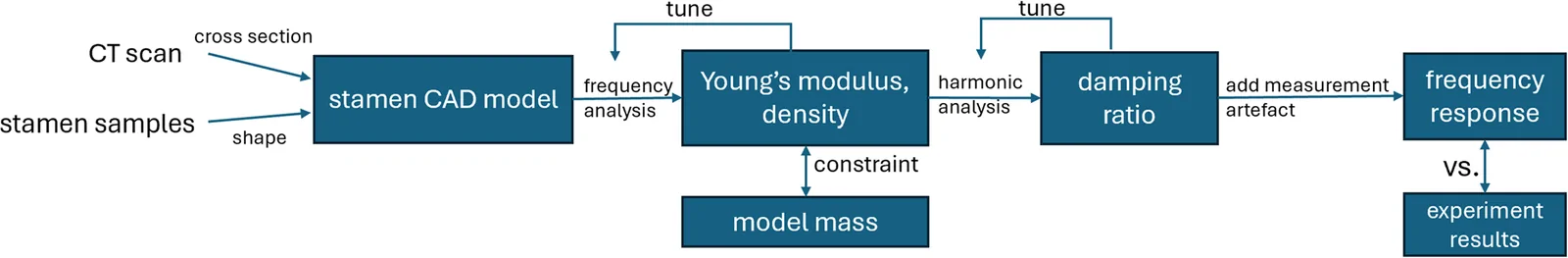
Workflow for constructing and tuning the FE model of the stamen. The stamen shape and cross-sectional geometry are obtained from CT scans and physical samples to build the CAD model. With a frequency analysis, the Young's modulus and density are tuned so that the resonance frequencies match with experimental results, subject to a total-mass constraint. With a harmonic analysis, the damping ratio is tuned to match the amplitude of each resonance. After incorporating the measurement artefact into the simulated response, the resulting transmissibility is compared with the experimental results.

As outlined in figure 8, the internal structure of the *M. magnifica* stamen is derived from CT scans of representative samples. However, the CT data are not directly converted into the geometry of the FE model, as during the relatively long scanning process, the stamen loses moisture and gradually straightens owing to drying (as shown in figure 12, but to a longer extent), resulting in a curvature different from that of the fresh samples used in the experiments. To preserve the experimentally relevant geometry, the stamen computer-aided design (CAD) model is reconstructed manually in SolidWorks (Dassault Systemès, Vélizy-Villacoublay, France, ver. 2021). The internal cross-sectional features are extracted from the CT data, while the overall curvature is based on photographs of the actual experimental samples.

**Figure 9.**
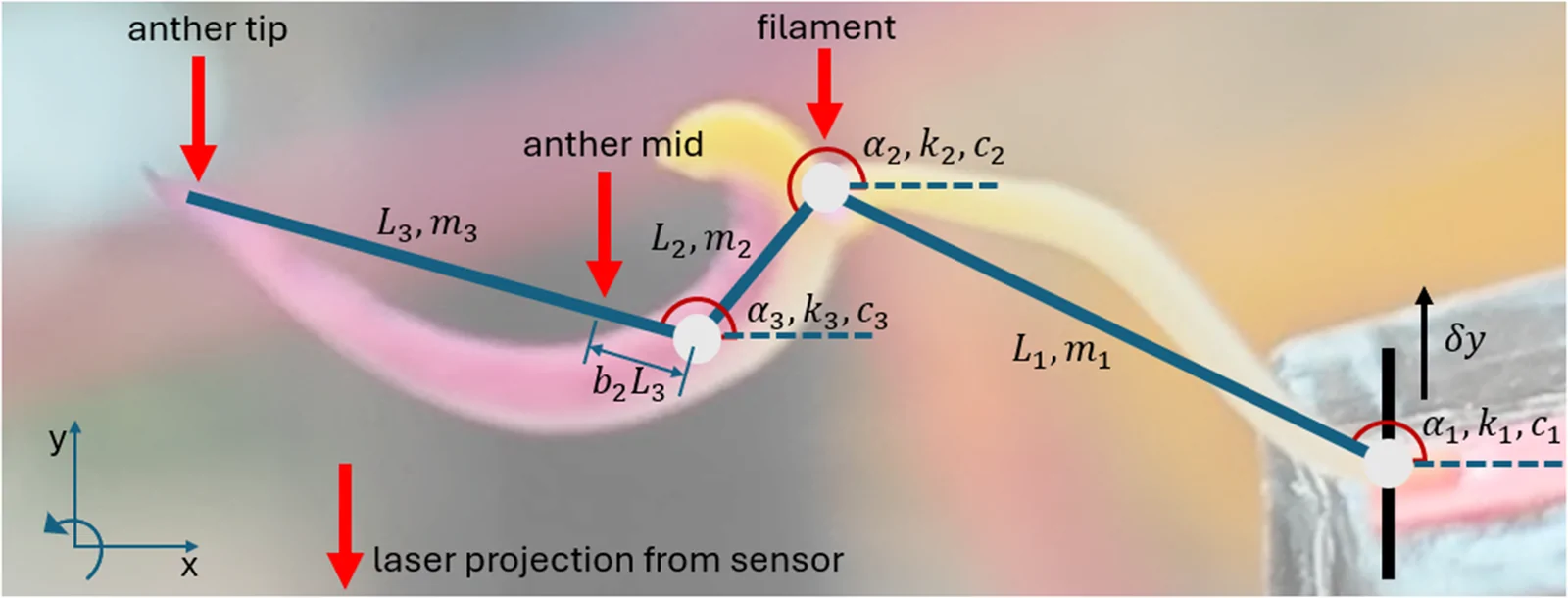
Schematic of the multi-body linkage model of an *M. magnifica* stamen superimposed on a photograph of the actual sample. The line–node combination illustrates the linkage system used to model the stamen. The length and orientation of each link are extracted from the photograph to preserve the natural curvature and proportions of the real stamen. The red arrow shows the laser alignment to the measurement locations.

**Figure 10.**
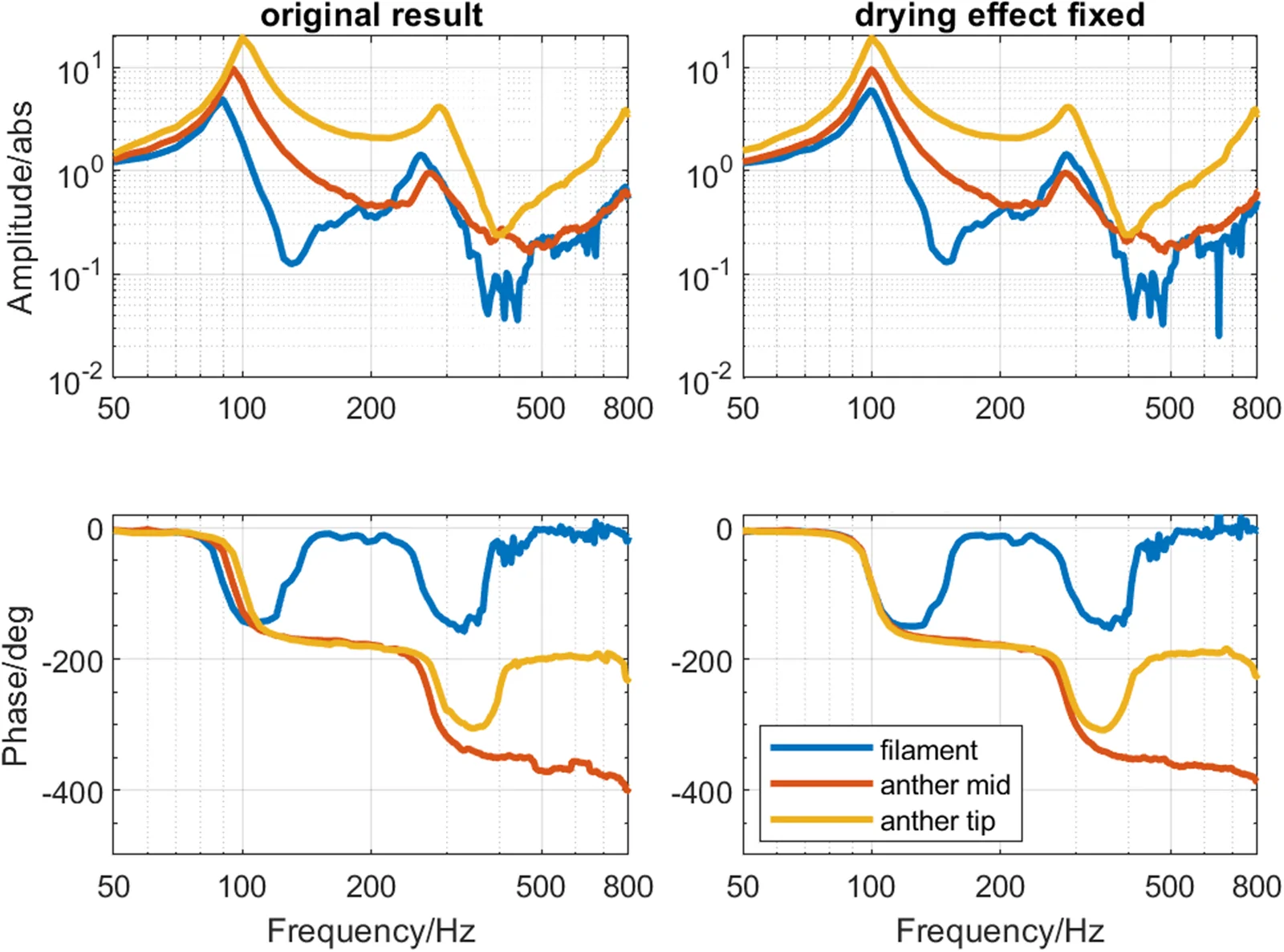
Transmissibility of an *M. magnifica* stamen before (left) and after (right) correcting for drying effects. Sequential measurements caused small downward shifts in resonance frequencies owing to water loss. To compare the dynamic response across locations, the first and second resonances of later measurements are matched to those of the first measurement as described in §2.6.

**Figure 11.**
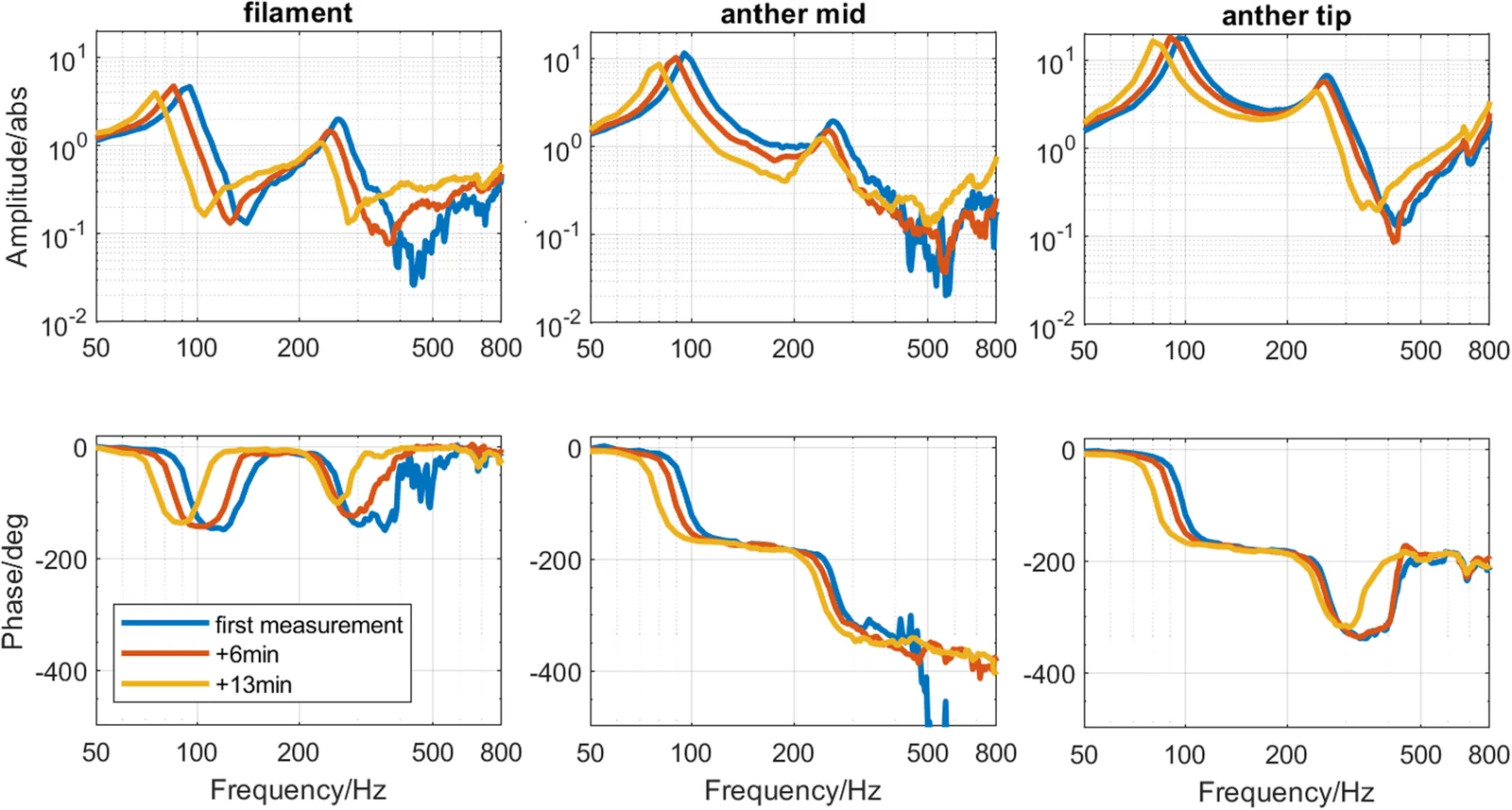
Transmissibility of an *M. magnifica* stamen sample measured at different times during drying. The resonance frequencies gradually shift to lower values as the stamen dries, and the amplitude of resonances decreases, demonstrating the influence of water loss on the dynamic response.

**Figure 12.**
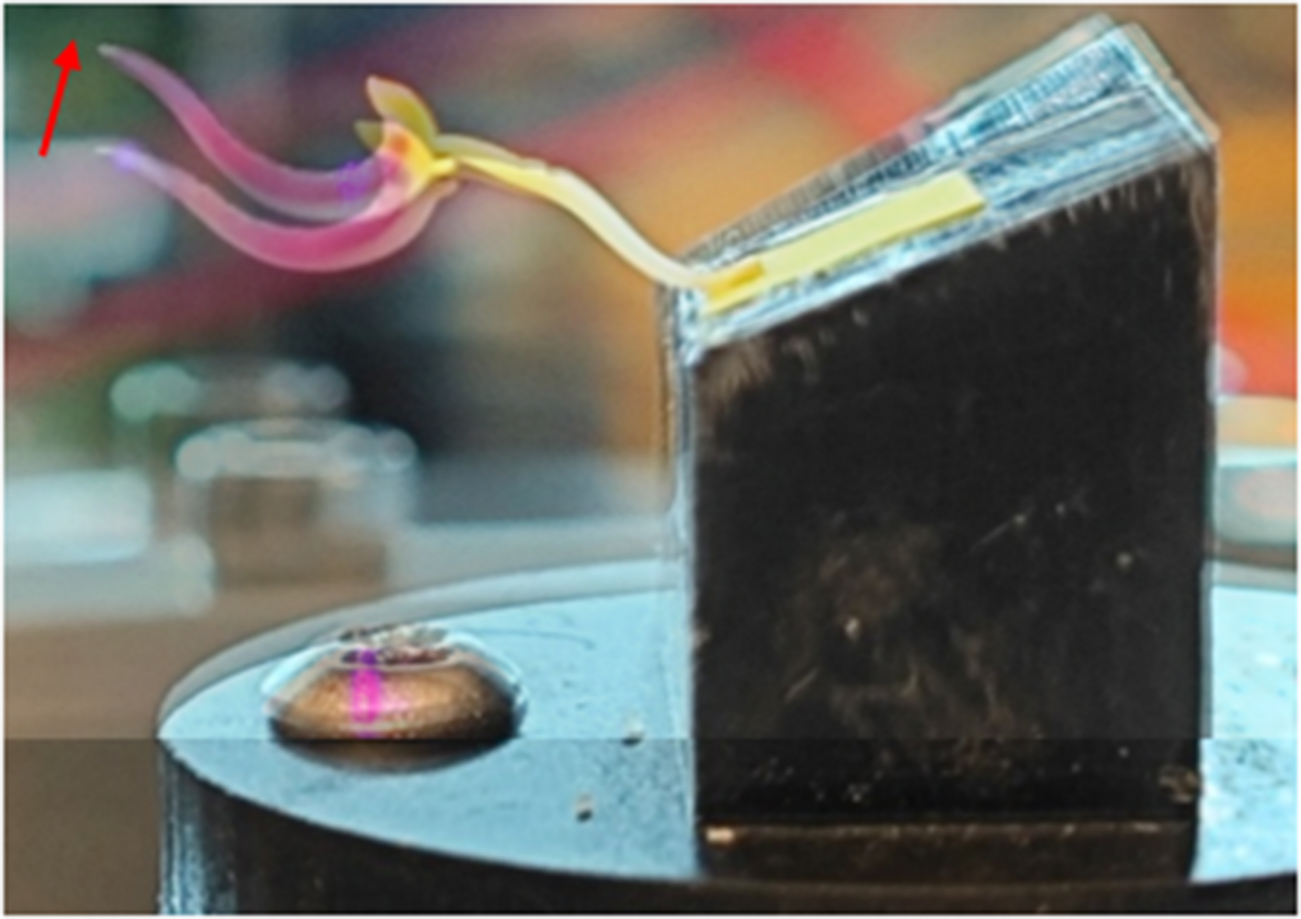
Overlapping photos of a stamen sample, one being taken when the sample was just placed on the stamen support, and the other taken 15 minutes after the first one. The arrow indicates the change of shape in 15 minutes.

The constructed model is divided into two material regions corresponding to the filament and the anther. This separation allows the assignment of distinct mechanical properties, reflecting the visible differences in colour, surface texture and tissue composition observed in the biological samples. Each region is assigned independent material parameters, which are subsequently adjusted in simulations to reproduce the resonances observed in the experiments.

The tuning of material properties is done in several steps with the FEA software Ansys (Ansys, Inc., Canonsburg, PA, USA, ver. 2025 R1), because the computation of the frequency analysis is fast but does not consider the damping effects, while the harmonic analysis provides a full dynamic response but requires a long computation time. To optimize the procedure, the Young's modulus and density of the materials are tuned, so that the frequency of each resonance predicted by the frequency analysis matches the experimental results. The damping ratios of materials are then tuned to match the amplitude of each resonance, obtained from the harmonic analysis of the FE model, to the experimental results. Finally, the amplitude and phase information of the harmonic analysis are used to calculate the transmissibility of the FE model and to synthesize the measurement artefact, which is compared to the experimental results for model validation.

### 2.9. Multi-body modelling

While the FE model provides detailed information on the stamen's motion and strain distribution, a multi-body model offers complementary insights into how physical properties of each subregion, such as mass, stiffness and damping, contribute to the overall dynamics. Moreover, the multi-body representation allows rapid modification of geometric parameters and provides excellent computational efficiency. It is therefore particularly suitable for investigating how variations in stamen geometry influence its dynamic behaviour.

Given the morphology of *M. magnifica* stamens, a linkage system with torsional springs (figure 9) is adopted instead of a conventional linear spring–mass model. The linkage formulation more accurately represents the coupled bending and rotational motions observed in the experiments. Although the mathematical description of a linkage system is more complex than that of a spring–mass model, the TMT method [25] is employed to derive the time-domain equations of motion (EoMs) efficiently, without explicitly calculating joint reaction forces. The EoMs are then linearized by assuming small angular displacements and transformed from the time domain into the frequency domain using the Laplace transform.

For the proposed linkage system, the mathematical expression of its transmissibility can be derived from the EoM as follows. The base motion is considered as an inertial force, and the generalized coordinates are defined as the variation of link orientations $\alpha_{1}$, $\alpha_{2}$ and $\alpha_{3}$:

$$q={\left[\begin{array}{ccc} \delta \alpha_{1} & \delta \alpha_{2} & \delta \alpha_{3} \end{array}\right]}^{T}.$$

The position of the centre of mass of each rod is

$$x_{m}={\left[\begin{array}{ccccccccc} x_{m1} & y_{m1} & \theta_{1} & x_{m2} & y_{m2} & \theta_{2} & x_{m3} & y_{m3} & \theta_{3} \end{array}\right]}^{T},$$

in which

$$\begin{array}{rl} x_{mi} & =\sum_{j=1}^{i}L_{j}\cos\theta_{j}-\frac{1}{2}L_{i}\cos\theta_{i},\\ y_{mi} & =\sum_{j=1}^{i}L_{j}\sin\theta_{j}-\frac{1}{2}L_{i}\sin\theta_{i}\\ \text{and}\;\theta_{i} & =\alpha_{i0}+\delta \alpha_{i}, \end{array}$$

where $\alpha_{i0}$ is the orientation of each link at the equilibrium state. Gravity is neglected, and the only external force acting on the system is the inertial force caused by the input base motion:

$$F_{y}={\left[\begin{array}{ccccccccc} 0 & -m_{1}\ddot{\delta y} & 0 & 0 & -m_{2}\ddot{\delta y} & 0 & 0 & -m_{3}\ddot{\delta y} & 0 \end{array}\right]}^{T}.$$

The passive elements are expressed as constraint terms:

$$c_{pi}=\delta \alpha_{i}-\delta \alpha_{i-1},\;\sigma_{i}=k_{i}c_{pi}\;\text{for springs}\text{ and}\;\sigma_{i}=c_{i}{\dot{c}}_{pi}\;\text{for dampers}.$$

The transformation and mass matrices are defined as

$$T=\frac{\partial x_{m}}{\partial q}\;\text{and}\;M=\mathrm{diag}[m_{1},m_{1},J_{1},m_{2},m_{2},J_{2},m_{3},m_{3},J_{3}].$$

The EoM can then be expressed as

$$T^{T}MT\;\ddot{q}=T^{T}\left(F_{y}-M\frac{\partial^{2}x_{m}}{\partial q\partial q}\dot{q}\dot{q}\right)-\sum_{i}\frac{\partial c_{pi}}{\partial q}\sigma_{i}.$$

The term $M\frac{\partial^{2}x_{m}}{\partial q\partial q}\dot{q}\dot{q}$ is neglected because $\dot{q}$ is small. Using the small-angle approximations $\sin(\alpha_{i0}+\delta \alpha_{i})\approx \sin\alpha_{i0}$ and $\cos(\alpha_{i0}+\delta \alpha_{i})\approx \cos\alpha_{i0}$, the EoM can be simplified as

$$A\ddot{q}=B\ddot{\delta y}+Cq+D\dot{q}.$$

Applying the Laplace transform yields

$$s^{2}\mathrm{AQ}=s^{2}B\delta Y+CQ+sDQ,$$

and thus the transfer function becomes

$$\frac{Q}{\delta Y}={\left(s^{2}IA-sID-C\right)}^{-1}s^{2}IB.$$

The vertical displacements at the filament, anther middle and anther tip are expressed as

$$y_{s}={\left[\begin{array}{c} L_{1}\sin\theta_{1}+1,\;\sum_{j=1}^{2}L_{j}\sin\theta_{j}+b_{2}L_{3}\sin\theta_{3}+1,\;\sum_{j=1}^{3}L_{j}\sin\theta_{j}+1 \end{array}\right]}^{T}$$

and the corresponding horizontal coordinates as

$$x_{s}={\left[\begin{array}{c} L_{1}\cos\theta_{1},\;\sum_{j=1}^{2}L_{j}\cos\theta_{j}+b_{2}L_{3}\cos\theta_{3},\;\sum_{j=1}^{3}L_{j}\cos\theta_{j} \end{array}\right]}^{T}.$$

By linearizing these expressions around the equilibrium position to get $\partial y_{s}/\partial q$ and $\partial x_{s}/\partial q$, and applying the Laplace transform to get $Y_{s}/Q$ and $X_{s}/Q$, the transmissibility from the excitation displacement at filament bottom to the displacement response at each measurement location is given by

$$\frac{Y_{s}}{\delta Y}=\frac{Y_{s}}{Q}\frac{Q}{\delta Y}+1\;\text{and}\;\frac{X_{s}}{\delta Y}=\frac{X_{s}}{Q}\frac{Q}{\delta Y}.$$

The displacement transmissibility obtained from the sensor read-out, which contains the measurement artefact, is

$$\frac{Y_{s}}{\delta Y}-\frac{X_{s}}{\delta Y}\cdot \tan\theta_{\text{local}}.$$

The relative length and equilibrium orientation of each linkage are extracted from annotated measurement points on a photograph of the individual stamen being modelled, and the model's transmissibility is matched to the measured results of the same sample. The mass of each linkage, as well as the stiffness and damping of each joint, is tuned so that the displacement transmissibility of the multi-body model closely matches that of the corresponding stamen from which the geometry is derived. The quality of the match is evaluated by comparing the two curves in terms of their resonance frequencies, resonance amplitudes and the overall shape of their transmissibility plot. Initially, each linkage is assumed to be a homogeneous rod, although this assumption can be refined if needed to achieve better agreement between the model and experimental data.

## 3. Results

### 3.1. Frequency response at different locations of *Medinilla magnifica* stamens

A typical transmissibility measured at three different locations along the stamen is shown in figure 10. Within the measured frequency range, the stamen exhibits two resonances. The response at the filament (blue) shows an anti-resonance following each resonance, while the response at the anther tip (red) shows an anti-resonance only after the second resonance. No anti-resonance is observed in the response at the anther mid (yellow).

Over 4 days during the blooming period of an *M. magnifica* plant, a total of 30 stamens from 15 flowers were measured. In many cases, the laser sensor could not reliably capture motion at the filament because the tissue in that region is more transparent and therefore less compatible with the optical sensor. Among these samples, 19 provided sufficiently high-quality data to calculate transmissibility at all three measurement locations. Of these, 15 samples displayed highly consistent patterns, similar to the example shown in figure 10. The upward trend near 800 Hz suggests that a third resonance likely exists slightly beyond the upper limit of the current measurement range.

The remaining four samples did not exhibit a clear anti-resonance after the second resonance at the filament or at both the filament and the anther tip. This absence of a detectable anti-resonance may indicate that these samples possessed slightly different dynamic properties, or that the motion amplitude at those locations became too small to be measured by the sensor after the second resonance.

### 3.2. Effects of stamen drying on measurement results

Figure 11 shows the effect of drying on the measured transmissibility of the stamen. At all three measurement locations, the first and second resonance frequencies decrease by approximately 10 Hz every 6 minutes of drying. In addition, the resonance amplitudes also decrease. These changes indicate that the dynamic response of the stamen is sensitive to water loss. Although a quantitative analysis of this effect is beyond the scope of the present study, the observation highlights the importance of carefully controlling or accounting for drying in similar dynamic measurements of biological samples.

It is not feasible to continue measurements beyond 15 minutes of drying because, as shown in figure 12, the stamen bends upward significantly as it loses water. This geometric deformation causes shadowing effects in the laser sensor, preventing it from detecting the laser points on the stamen surface.

### 3.3. Finite-element model

**Figure 13.**
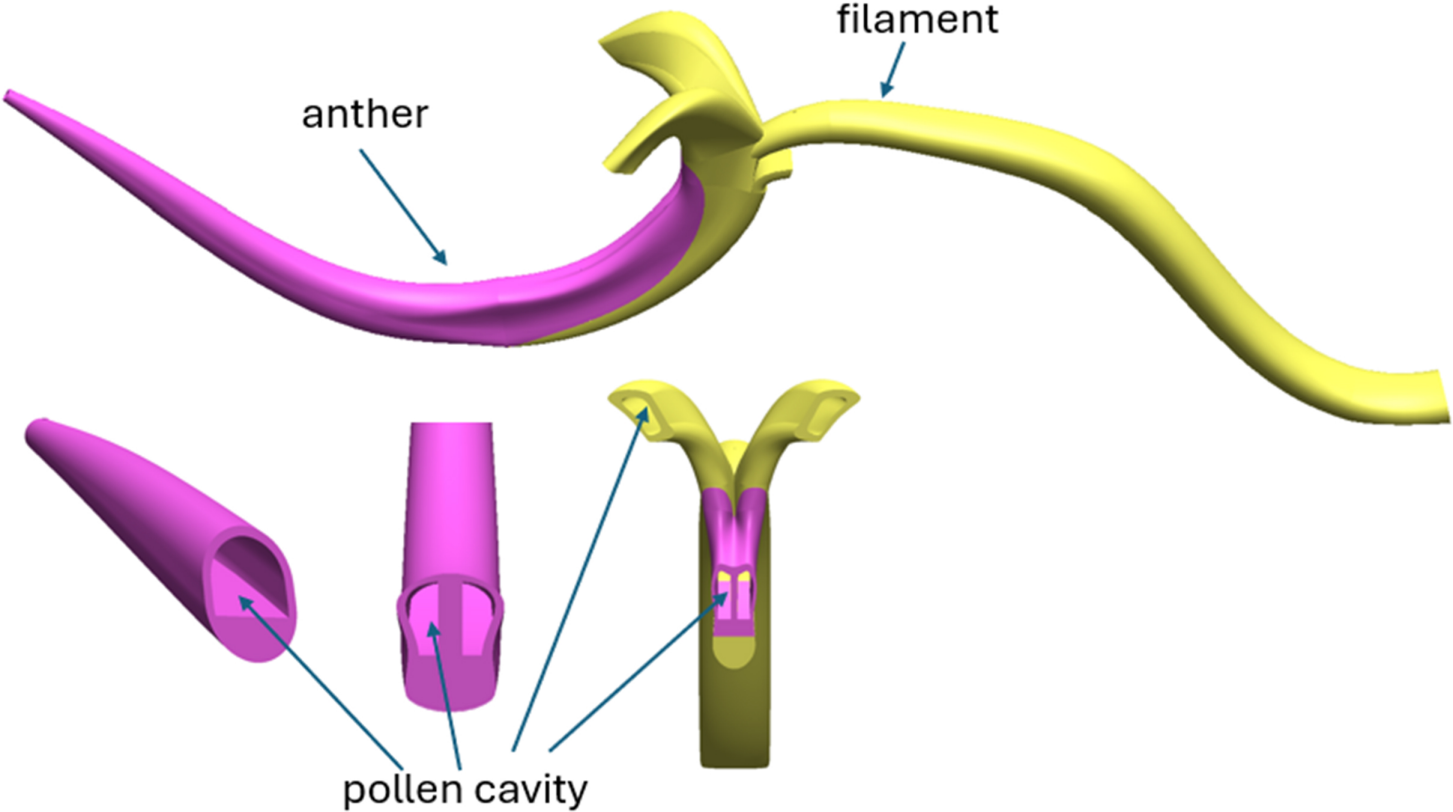
CAD model of the stamen. The geometry of the cross-section varies along the anther. In the middle and bottom regions of the anther, the pollen cavity is divided by a central wall. In the tip region of the anther, the two halves merge into one cavity. The anther and filament are marked in purple and yellow individually, similar to real stamens.

The CAD model of the stamen is shown in figure 13. The model consists of two parts—the anther (purple) and the filament (yellow)—assembled together. The material parameters of these two parts, listed in table 1, are obtained by tuning the Young's modulus, density and damping ratio in the FE model until the resonance frequencies and amplitudes match the experimental results of real stamen samples. The Young's modulus is in the reported range of plant tissue [26]. The stamen is constrained at the bottom of the filament to represent its attachment to the shaker in the experiments.

**Table 1.** Material properties of the FE model.

	anther	filament
Young's modulus (MPa)	28	28
density (kg ${\text{m}}^{-3}$)	700	700
damping ratio	0.1	0.06

**Figure 14.**
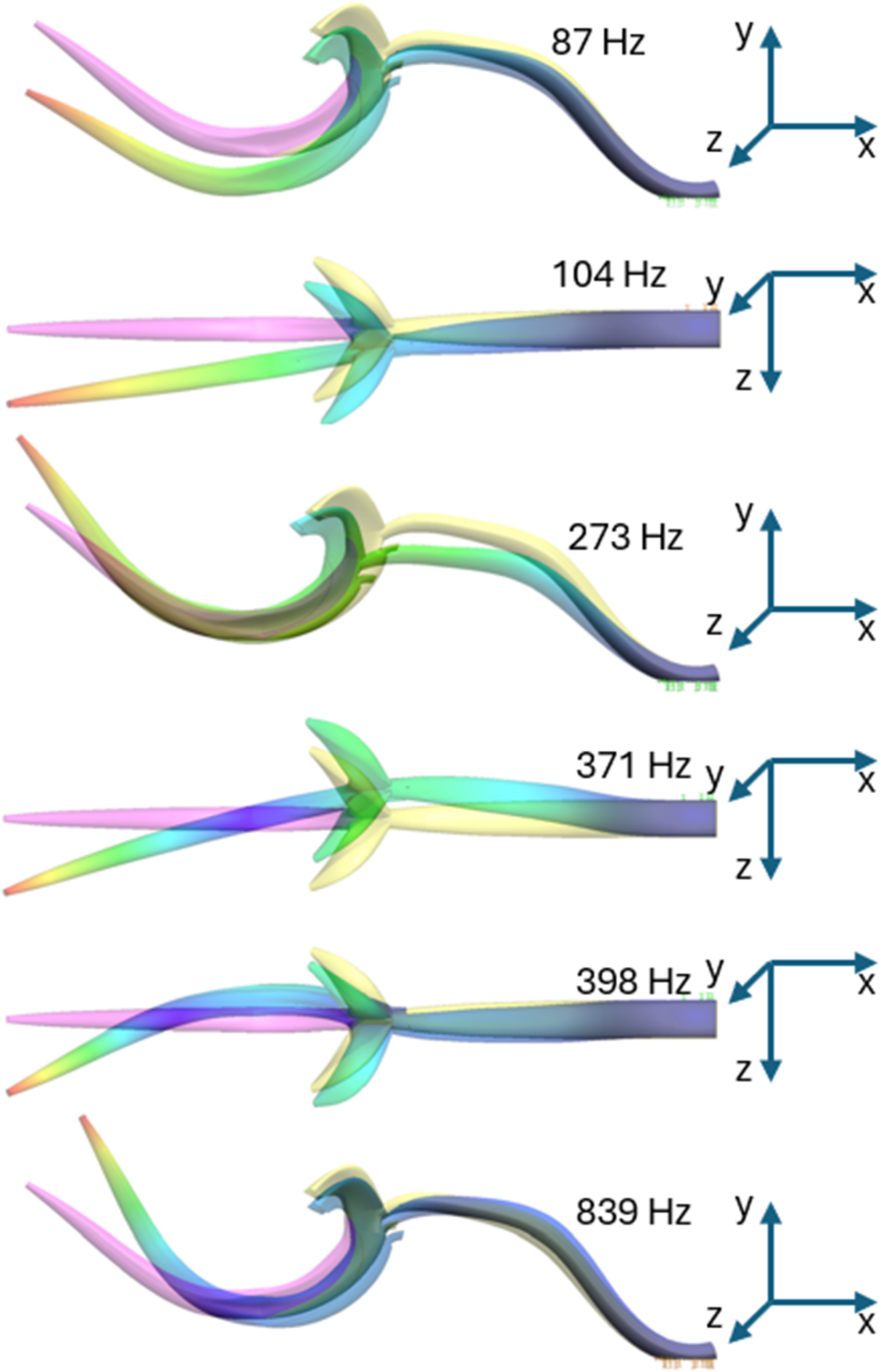
All structural modes of the FE model are below 1000 Hz. The first, third and sixth modes are motions in the x–y plane. The second and fourth modes are motions in the x–z plane. The fifth mode is a torsional motion about the x-axis. For clearer visualization, these are generated in SolidWorks frequency analysis. Resonance frequencies and mode shapes are cross-checked with Ansys.

All modes below 1000 Hz for this stamen model are shown in figure 14. The first and second resonances in the experimental results correspond to the first and third modes, occurring at 87 and 273 Hz, respectively, both within the range of the experimentally measured resonances. The second, fourth, and fifth modes are not observed in the experiments, as they require excitation in the z-direction or torsional excitation. The sixth mode is at 839 Hz, and agrees well with the former speculation that there is a third resonance slightly beyond 800 Hz. This model is therefore used for the subsequent harmonic analysis.

**Figure 15.**
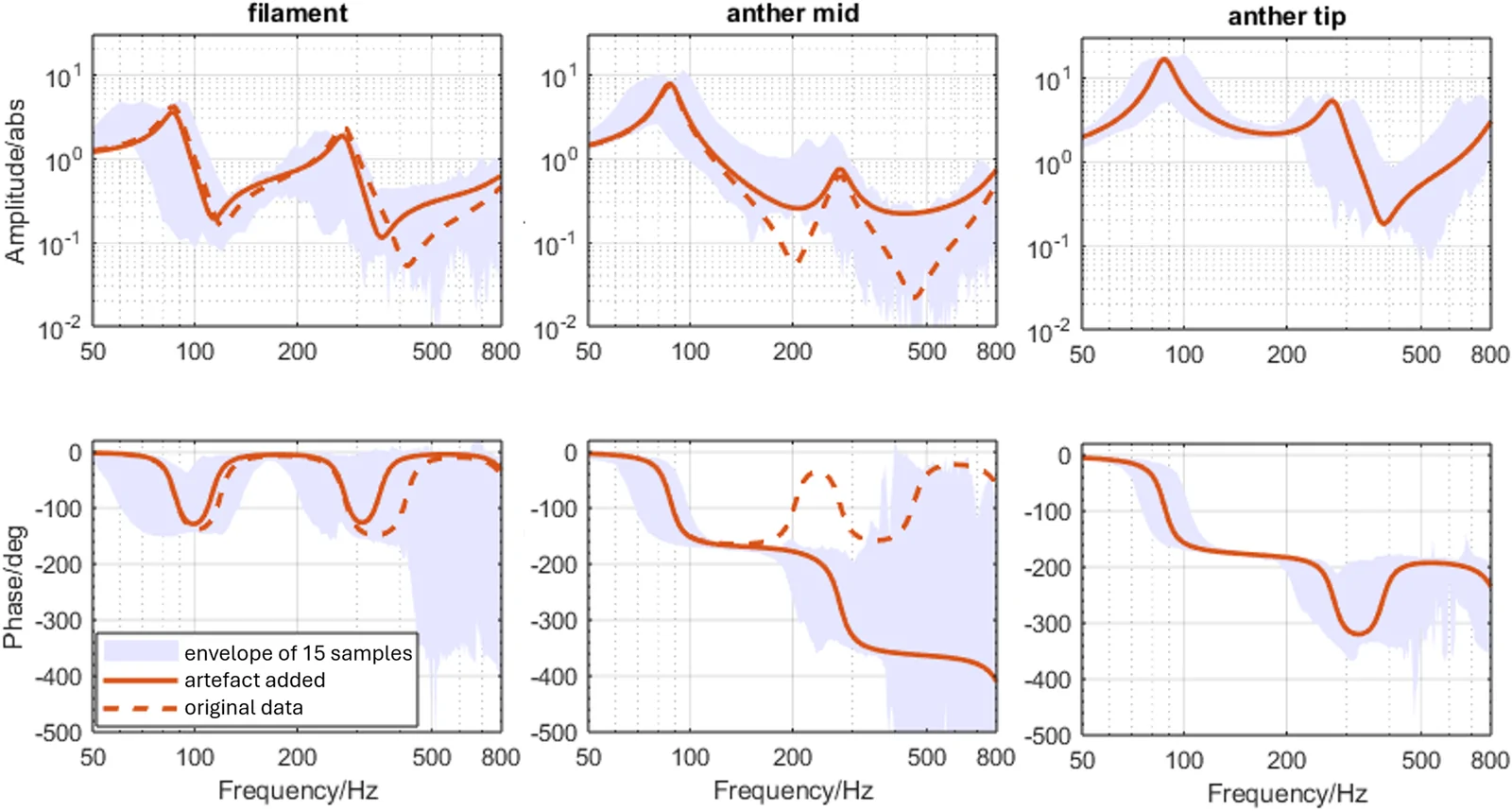
Transmissibility of the FE model, obtained from Ansys harmonic response. The shaded area is the envelope of the transmissibility at each measurement location of the 15 stamen samples (the data of filament and anther mid here are not fixed for drying effect, so the resonances they show are at slightly lower frequencies than the anther tip data show). At the filament and the anther mid, a better coherence between the model and samples requires adding the measurement artefact.

From the harmonic analysis of the FE model, the displacement transmissibility from the filament base to the three measurement locations is obtained in the x- and y-directions. To ensure comparability between simulations and experiments, the measurement artefact described in §2.7 is incorporated into the simulated transmissibility. This artefact accounts for the laser sensor's limited ability to follow the true nodal motion owing to local surface slope and x-direction drift. As shown in figure 15, the simulated transmissibility at the filament and at the anther tip falls well into the shaded envelope representing the transmissibility of the 15 measured samples, while the transmissibility at the anther mid deviates significantly from the envelope in both amplitude and phase response. When the artefact is included, all simulated transmissibility falls well into the shaded envelope, particularly at the anther mid, confirming that this correction captures an essential aspect of the experiment.

### 3.4. Multi-body model

After empirically tuning the mass, torsional stiffness and damping of each link and joint to the values listed in table 2, the transmissibility of the multi-body model is compared with the experimental result of the stamen sample from which the link dimensions and orientations are extracted. The model parameters are adjusted so that the resonance frequencies and amplitudes match those measured experimentally. The measurement artefact is then incorporated into the model by superimposing the synthetic artefact signal onto the simulated displacements.

**Table 2.** Parameters of the dimensionless linkage system. $b_{2}=0.11$, local slope at anther mid is 20°.

link/joint no.	length $L_{i}$	orientation $\alpha_{i}$	mass $m_{i}$	stiffness $k_{i}$	damping $c_{i}$
1	$19.8\times 10^{-3}$	151	$1.8\times 10^{-6}$	$9\times 10^{-4}$	$1.5\times 10^{-7}$
2	$7.7\times 10^{-3}$	−141	$2\times 10^{-6}$	$3.6\times 10^{-4}$	$1.6\times 10^{-8}$
3	$15.3\times 10^{-3}$	163	$0.8\times 10^{-6}$	$6\times 10^{-4}$	$1\times 10^{-8}$

**Figure 16.**
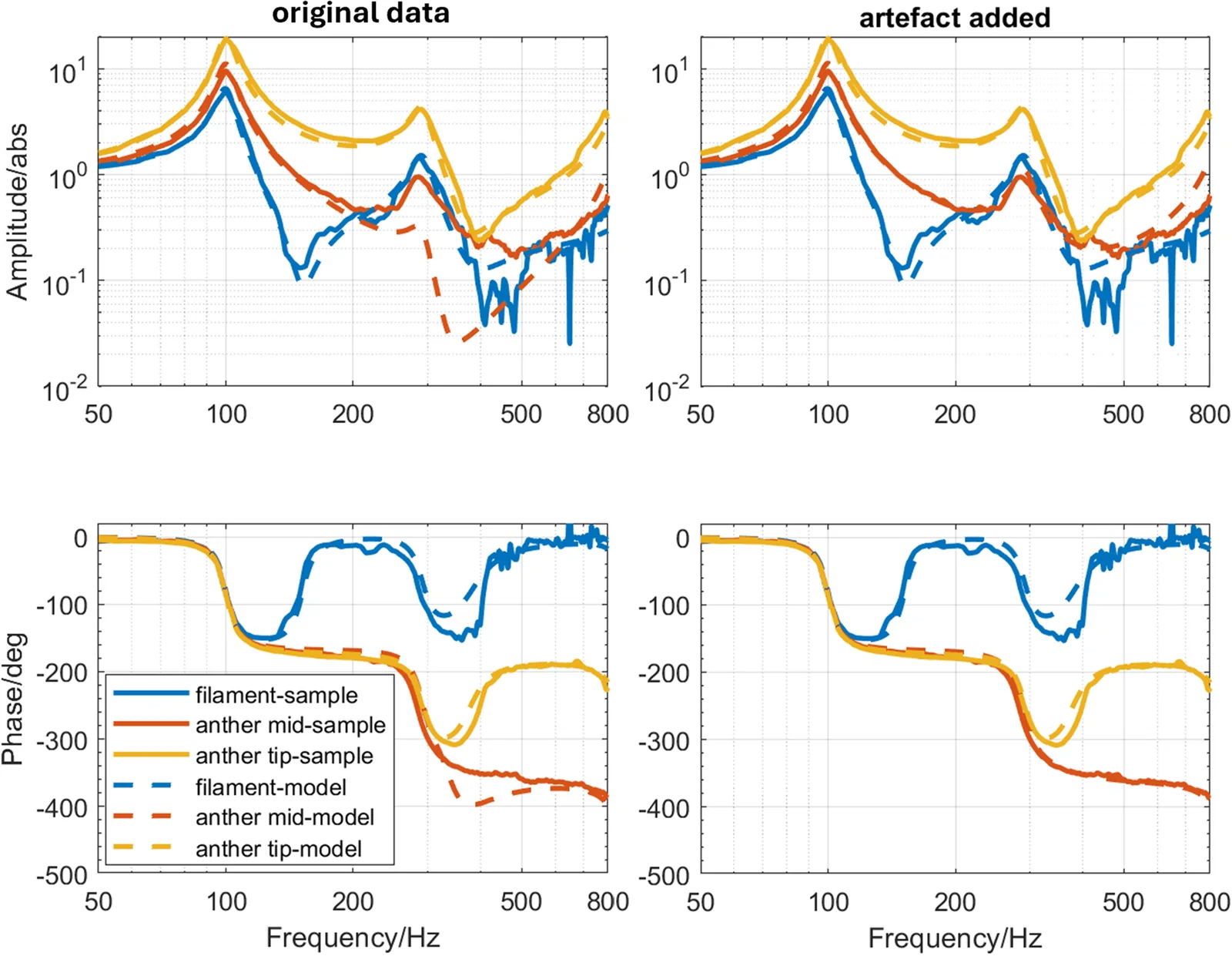
Transmissibility of the multi-body model comparing to the sample (here the filament and anther mid data are drying effect fixed). Adding the synthesized measurement artefact at the anther mid significantly improves the match to the transmissibility of the real stamen sample.

The transmissibility of the linkage system is shown in figure 16. Without including the artefact, the transmissibility at the anther tip and filament agrees well with the experimental result, whereas the transmissibility at the anther mid shows noticeable deviation. When the artefact is included, the transmissibility at all three locations closely matches the experimental result.

To demonstrate the capability of the multi-body model in studying the influence of subregional properties on the dynamics of stamen, three supplementary studies are presented in the appendices. Appendix A in the supplementary material shows that each joint stiffness primarily governs one resonance frequency. Appendix B in the supplementary material establishes that the three joint stiffnesses are uniquely identifiable from the three measured resonance frequencies when link masses are fixed. Appendix C in the supplementary material uses three additional specimens to show that some dynamic features originate from the stamen geometry.

## 4. Discussion

### 4.1. Dynamic behaviour of *Medinilla magnifica* stamens and implications for buzz pollination

The presented experiments and models reveal three resonances of the *M. magnifica* stamen under 1000 Hz in the vertical direction. The first mode lies slightly below the reported frequency range of pollination buzz (100–400 Hz), the second falls within this range and the third occurs about 300–500 Hz above this range. The structural resonances of the stamen give the possibility that a bee can excite large motion of the stamen with small effort, if it can buzz at a resonance frequency of the bee–stamen system. However, current results cannot predict which resonance a bee could use, although the measurement shows that only the second resonance is in the frequency range of buzz pollination. The body mass of a bee will shift down the resonance frequencies of the bee–stamen system during pollination [16]. From a mechanical perspective, the flexibility of the bee's thorax and joints may also change the resonance frequencies of the bee–stamen system and the mode shape of each resonance.

Furthermore, it is worth speculating that efficient pollen release may depend not only on the global motion amplitude of the stamen but also on its internal deformation pattern. For continuous pollen release to occur, pollen needs to travel from the base of the anther to the pore at the tip, a process which likely relies also on the internal deformation of the anther rather than only on the global motion amplitude. A change of the internal deformation pattern with frequency is suggested by the phase difference between each region of the stamen, which is the consequence of local anti-resonances instead of resonances. Accompanying the first anti-resonance of the filament, the phase plot shows that a phase difference of 180° appears between the filament and the anther (anther tip and anther mid). This phase difference means that beyond this frequency, the filament and the anther move in opposite directions. Beyond the second resonance, this phase difference shifts so that the anther tip moves opposite to the rest of the stamen. This opposite motion of the anther tip could, in principle, facilitate the transport of pollen from the base of the anther towards the apical pore. However, this hypothesis remains untested and would require direct observation of pollen motion within the anther to confirm.

In the mechanical aspects, the phase difference between each region is a sign of ‘motion decoupling’ of the stamen, indicating the redistribution of energy. In an efficient buzz pollination process, energy should be channelled within the anther to mobilize the pollen inside, without being spread to the filament. Furthermore, when a small bee is not strong enough to mobilize the entire anther, it still has a chance to extract the pollen located in the tip region of the anther. The mode shape of the sixth mode in figure 14 supports this scenario with a significant deformation at the anther tip, whereas deformation in the middle of the anther and the filament is barely visible. This regional deformation of the anther could be a possible explanation for the common phenomenon in Melastomataceae stamens that small bees buzz the anther tip when they are likely not strong enough to excite the entire anther (B. Lazarus and A. Dellinger 2025, personal communication) or not large enough to bite further while keeping contact with the anther pore [16].

Taken together, the presented results indicate that efficient pollen extraction may not require bees to tune their buzz frequency precisely to a particular stamen resonance. Instead, pollination could rely on a broader frequency region within which the anther is excited into deformation patterns that effectively mobilize pollen. This perspective provides a useful context for previous findings showing that floral sonication frequency does not vary systematically with bee body size [27], despite the common assumption that smaller bees compensate for their lower mass and strength by buzzing at higher frequencies than large bees. If small and large bees of the same lineage achieve pollination success with similar floral-buzz frequencies, then frequency alone is unlikely to be the main determinant of pollen release. Any size-related differences in buzz pollination efficiency may therefore arise from other features of bee–stamen interaction.

### 4.2. Comparison with *Solanum* stamens

**Figure 17.**
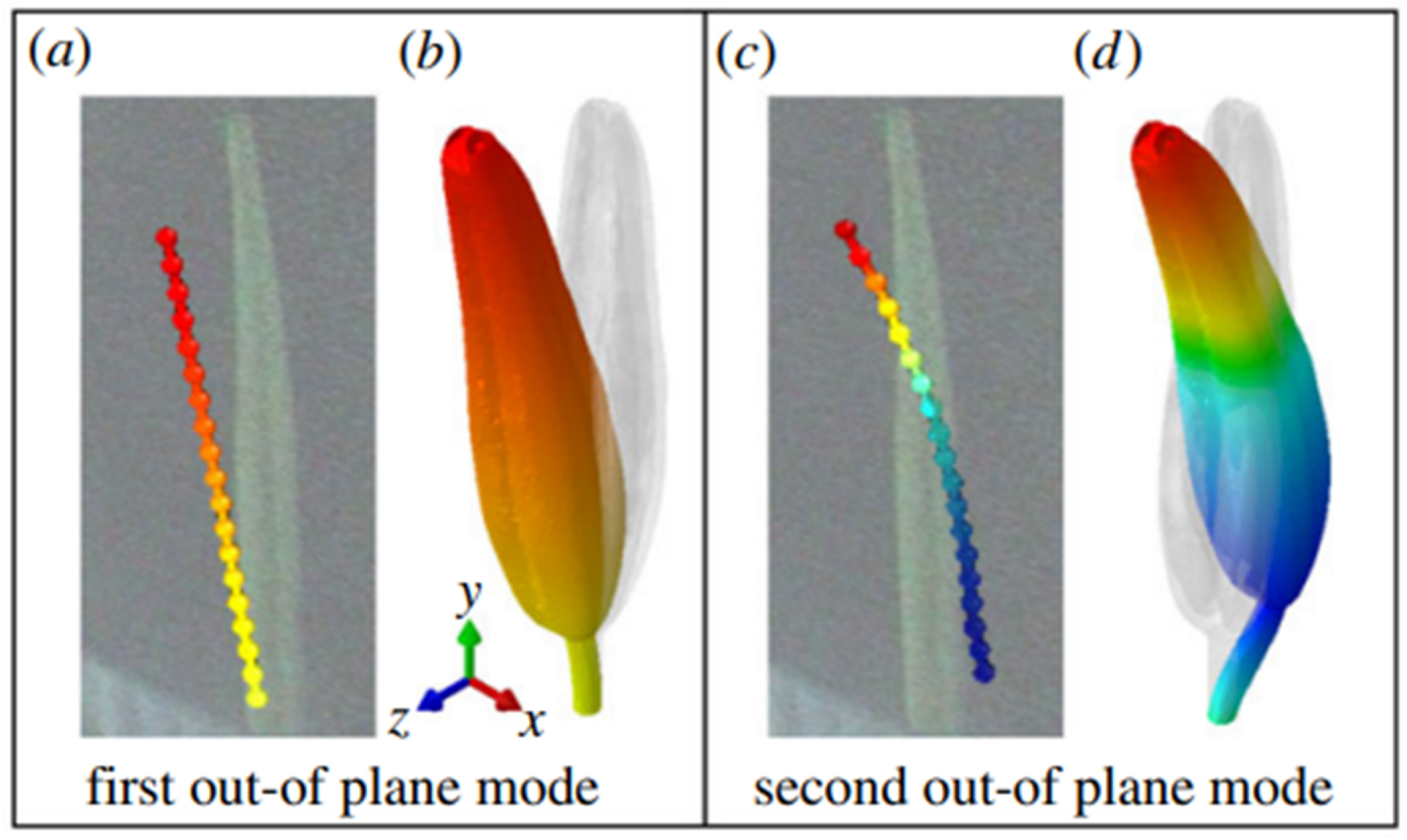
The two out-of-plane modes of *Solanum* stamen within 2000 Hz. (*a*,*c*) Measured shapes. (*b*,*d*) FEA results. In the FEA, the first mode is at 64.2 Hz and the second one is at 1234.4 Hz. Reproduced from Jankauski *et al.* [15], fig. 5 (CC-BY).

The stamens of *M. magnifica* exhibit far more complex dynamic behaviour than those reported for *Solanum* taxa. First, the feeding anthers of *Solanum fructu-tecto* and *S. elaeagnifolium* have been reported to show only a single resonance within 1000 Hz [14,15], in contrast to the three resonances identified in *M. magnifica*. Second, FEA of *Solanum* stamens shows that all out-of-plane modes up to 2000 Hz (shown in figure 17) involve little deformation of the anther tube, and the structure behaves largely as a rigid body pivoting about the filament base. By contrast, in *M. magnifica*, the mode shapes from the third mode in figure 14 onward exhibit substantial anther deformation. In general, if bees exploit the dynamic behaviour of the stamen, the *M. magnifica* stamen provides a broader range of mechanically distinct modes to excite, and the elasticity of its anther may directly contribute to pollen release.

This complexity likely arises from the more elaborate morphology of the *M. magnifica* stamen. Compared with the stamens of most *Solanum* species, *M. magnifica* possesses a longer filament and a more slender anther, resulting in its second and third resonances occurring at relatively low frequencies. In addition, like many other members of the Melastomataceae, the anther of *M. magnifica* is strongly curved, whereas most *Solanum* anthers are nearly straight. This curvature likely explains why the *M. magnifica* stamen exhibits an anti-resonance at the anther tip following the second resonance, a feature not typically observed in *Solanum* stamens.

Taken together, these differences suggest that the pollen-release mechanisms of *M. magnifica* and other Melastomataceae species likely diverge from those of *Solanum*, potentially suggesting that the family Melastomataceae selects its pollinators through different interaction patterns and shows distinct evolutionary adaptation.

### 4.3. Significance of the presented method

The experimental measurements of *M. magnifica* stamens provide sufficient information to construct and validate numerical models of the stamens. By comparing the measured displacement transmissibility with that obtained from simulations, the experimental results serve not only as direct observations but also as an effective benchmark for assessing model accuracy. After incorporating the synthesized measurement artefact caused by dynamic laser-spot drift along the stamen, the transmissibility predicted by the FE model and the multi-body model closely matches the experimental results. This agreement confirms that the modelling method accurately captures the essential dynamic behaviour of the stamen and can therefore be used with confidence for further investigation of buzz-pollination dynamics. For example, the computational models allow mode shapes, internal deformation patterns and anti-resonance behaviour to be examined in detail, including frequencies at which the experimental amplitude becomes too small to measure reliably. Besides, the improved agreement obtained after accounting for the measurement artefact indicates that the dominant systematic error in the experimental measurement of stamen motion has been successfully identified. Recognizing and compensating for this artefact is therefore essential for future studies of stamen dynamics and for developing reliable experimental protocols in buzz-pollination research.

The multi-body model complements the FE model by providing insights that are difficult to obtain from FEA alone. Because each parameter of the multi-body model corresponds to a distinct physical property of one subregion, the influence of individual properties can be isolated directly. For instance, it reveals that each resonance frequency is primarily governed by the local stiffness of one subregion, and that the filament contributes to the dynamics through its compliance rather than its inertia (supplementary material, appendix A). Furthermore, the model's computational efficiency and ease of geometric modification enable rapid cross-specimen analysis, showing that the qualitative transmissibility topology is governed by geometry while the resonance frequencies are determined by subregional stiffness (supplementary material, appendix C). Such analyses would require manual CAD reconstruction and repeated meshing and computation in the FE framework, making them impractical for comparative studies across specimens or species.

### 4.4. Advantages and limitations of the proposed measurement and modelling method

**Figure 18.**
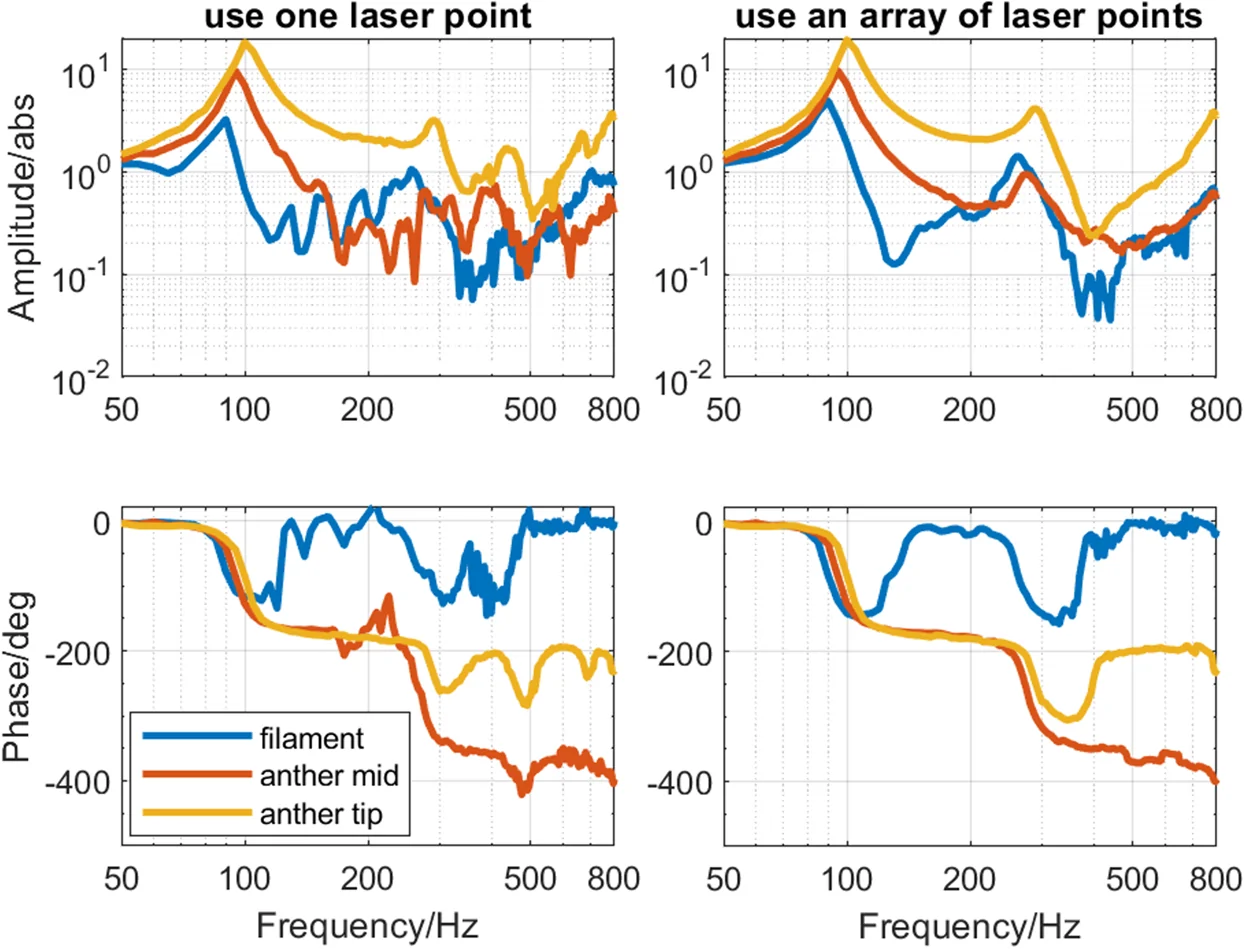
Comparison between the transmissibility of the same stamen sample obtained from a single laser point and from the full laser-line array. The laser-line measurement produces smoother and more consistent results, while the single-point measurement captures only the first resonance clearly.

Several specific choices in the methodological design contribute to the robustness of the results. The laser-line triangulation sensor provides high-quality, spatially resolved measurements with sufficient sensitivity to identify multiple resonances and anti-resonances, enabling a detailed experimental characterization of the stamen's vibration response. To illustrate the effect of improving measurement quality by using a laser-line sensor, the measurement data are reprocessed to simulate the output of a laser point sensor. For each frequency sweep, the displacement signal recorded at a single pixel position of the laser line is extracted and treated as the response of a point sensor. The resulting transmissibility, shown in figure 18 (left), exhibits noticeable irregularities and local fluctuations in both amplitude and phase, obscuring the second resonance and the anti-resonances. These distortions arise partly from the anther's motion in the z-direction, which causes the laser spot to drift across the surface, and partly from variations in the stamen's surface properties.

When the transmissibility is calculated using the full array of laser points (figure 18, right), these irregularities are largely suppressed because the laser-line sensor effectively averages signals over multiple adjacent points. This spatial averaging enhances robustness against surface effects and the drift induced by z-direction motion, confirming that the use of a laser-line sensor provides a more stable and representative measurement of the stamen's dynamic response.

The multi-body model, although rarely used in floral biomechanics, proves to be particularly valuable. It is computationally lightweight, easy to modify and compatible with open-source tools. It offers direct insight into how the dimensions, mass, stiffness and damping of each structural subregion give rise to the observed transmissibility. Its reduced number of degrees of freedom makes it particularly suitable for exploring how specific morphological features influence the overall dynamic behaviour.

The limitation of the proposed method is that the experiments and numerical models are validated only within the linear range. In real buzz pollination, the stamen displacement can reach several millimetres, which exceeds the measurement range of the laser-line sensor. Moreover, large deformations of the stamen can introduce shadowing effects that further limit sensor performance. To address this limitation, future development of the numerical models may focus on fully capturing the nonlinear behaviour of the stamen. Geometric nonlinearity could, in principle, be represented by replacing the linearized frequency-domain conversion with time-domain integration. However, material nonlinearity remains unresolved, as the nonlinear mechanical properties of plant tissues are currently not well characterized.

In summary, this study presents an integrated experimental–computational framework for characterizing the structural dynamics of poricidal anthers. The combination of high-resolution vibration measurements with validated FE and multi-body models shows that the anther's dynamic behaviour can be captured with high fidelity. The models provide an efficient and accurate tool for computational analysis, enabling exploration of how morphological and loading factors influence vibrational response. Together, these results establish a robust basis for future studies of buzz pollination and facilitate comparative analyses across morphologically diverse taxa.

## Supplementary Material



## Data Availability

The datasets supporting this article are available in the Zenodo repository [28]. Supplementary material is available online [29].
