# Supplementary material for: Measuring and modelling the structural dynamics of poricidal anthers: a mechatronic approach
Source: J R Soc Interface. 2026 Sep 9;23(242):20251321. doi: 10.1098/rsif.2025.1321 (PMC13561079; doi:10.1098/rsif.2025.1321)
Supplement: Supplementary file 1 [file rsif.2025.1321.zip › rsif.2025.1321/rsif20251321_si_001.pdf]

APPENDIX

A. Sub-regional parameter study with the multibody model

Here we use the multibody model to investigate which sub-regional properties of the stamen determine its structural dynamics. With the model parameters shown in Table II of the paper as a reference, we first halve the sub-regional mass respectively.

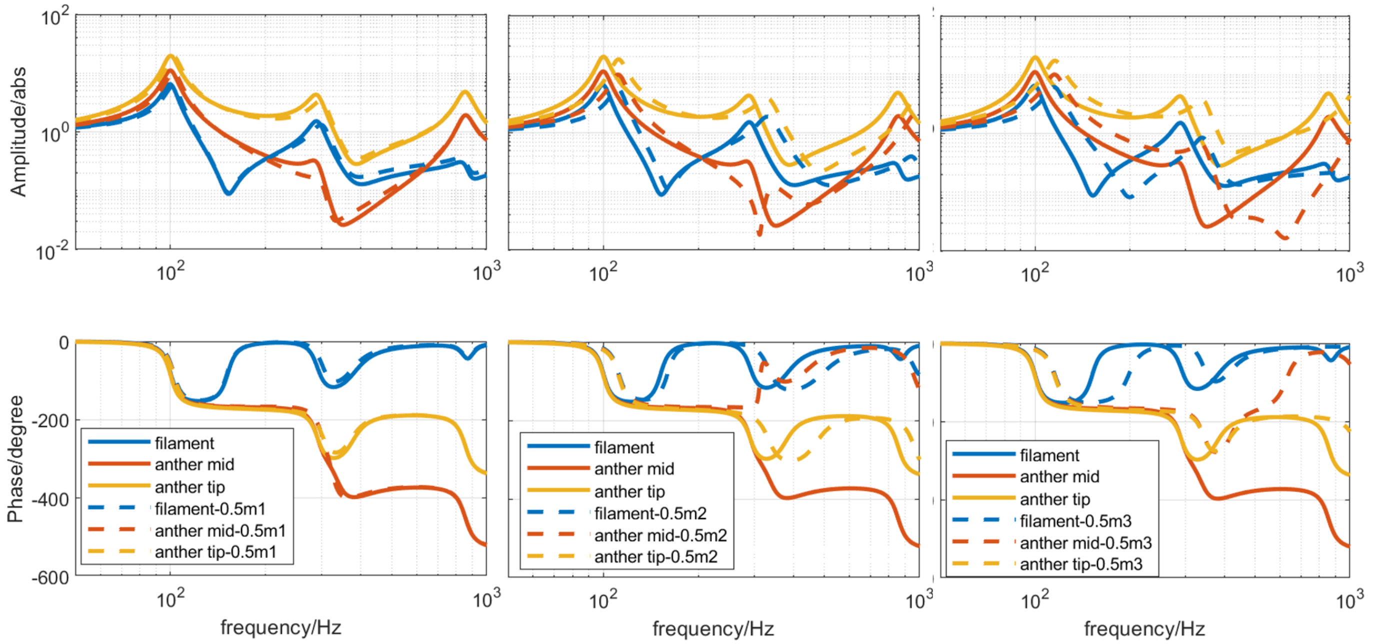

Fig. 19: Halving m1 (the mass of link 1) very slightly shifts up all resonances of the model. Halving m2 and m3 both moderately shift up the first two resonances, while halving m3 shifts up the 3rd resonance more.

Halving each link mass individually shows that reducing the filament mass (m1) has negligible effect on the resonance frequencies, whereas reducing the anther mid mass (m2) or anther tip mass (m3) shifts both the first and second resonances upward. This indicates that the filament functions primarily as a compliant transmission element whose contribution to the dynamics is through its stiffness rather than its inertia, while the anther provides the dominant inertial contribution.

Then, we halve the joint stiffness respectively. It should be noted that the sub-regional stiffness in the multibody model is not equivalent to the Young's modulus in the FE model. The joint stiffness reflects the combined effect of material stiffness and local cross-sectional geometry, including wall thickness and cavity shape. Two stamens with identical tissue material could therefore have different joint stiffnesses if their cross-sectional morphologies differ.

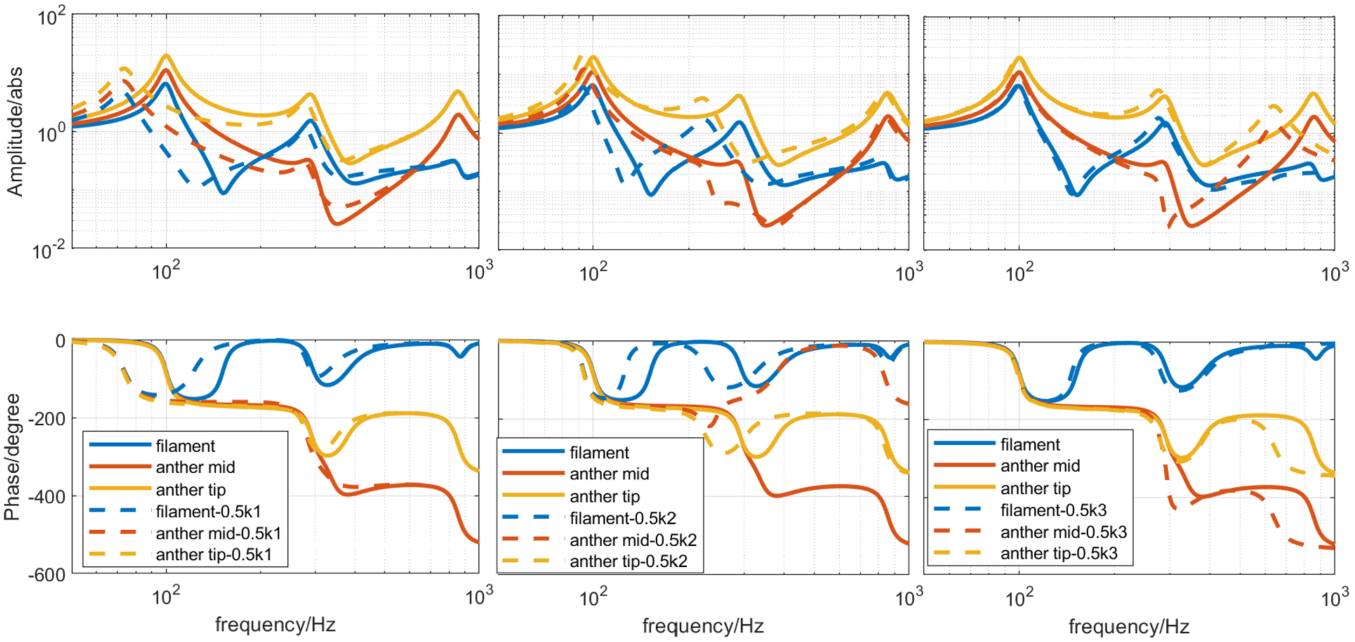

Fig. 20: Halving k1 (stiffness of joint 1) significantly shifts down the 1st resonance and slightly shifts down the 2nd resonance, while has no visible change on the 3rd resonance. Halving k2 significantly shifts down the 2nd resonance, while slightly shifts down the 1st and 3rd resonance. Halving k3 significantly shifts down the 3rd resonance and slightly shifts down the 2nd resonance, while barely affects the 1st resonance.

Halving each joint stiffness individually shows that each stiffness primarily governs the frequency of one resonance with only weak coupling to the other resonances. This observation is coherent with the experimental results that a specimen with the 1st resonance at a relatively high frequency does not necessarily have the 2nd or the 3rd resonance at a relatively high frequency, suggesting the stamen of Medinilla *magnifica* have relatively independent dynamic regions.

Last, to assess the role of geometry independently, the link lengths and orientations are extracted from photos of different specimens while all link mass, joint stiffness, and joint damping are held at the original values.

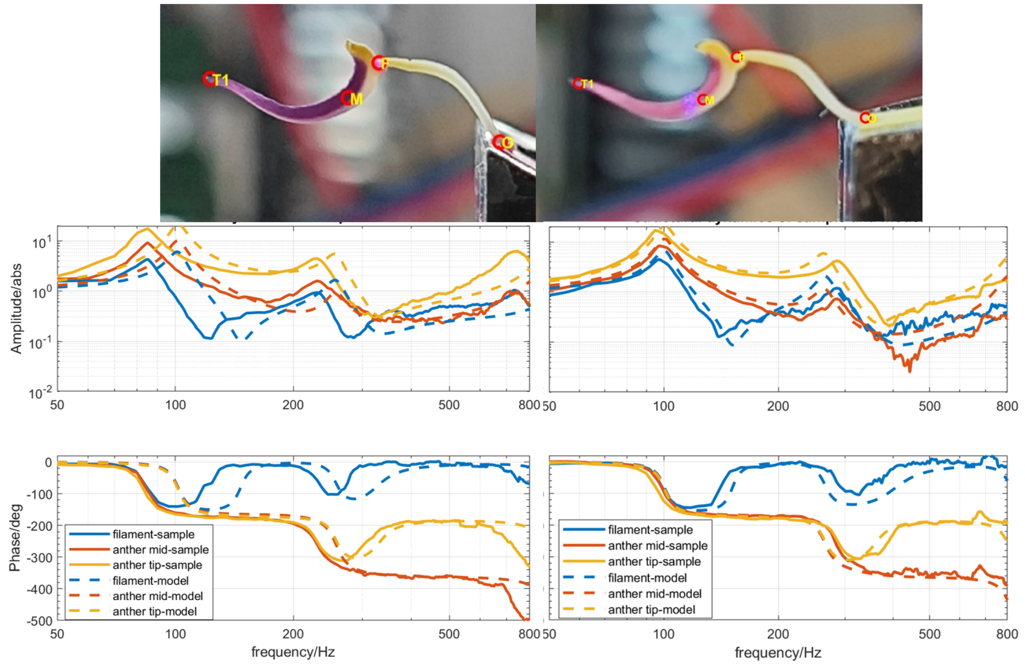

Fig. 21: The photos of two other specimens and their experimentally measured transmissibility (here the filament and anther mid data are drying effect fixed), and the modelled transmissibility with only geometry updated. None of the resonances match with experimental results.

The resulting resonance frequencies deviate from those measured experimentally for that specimen, suggesting that geometry alone cannot account for the specific resonance frequencies of individual specimens without corresponding adjustment of the sub-regional stiffnesses. Combined with the results of halving joint stiffness, we raise the hypothesis that the variation in resonance frequencies primarily originates from the variation in sub-regional stiffness, which is usually affected by cross-section dimension and tissue hydration status.

B. Stiffness identifiability study of the multibody model

By investigating the equation of motion, it is clear that with given model geometry and linkage mass, when the frequencies of the first three resonances in x-y plane are known, the value of (k1 k2 k3) can be found with the following procedure.

The displacement transmissibility of the multibody model is derived from the linearised equations of motion as described in the method section. The transfer function from the base excitation δy to the generalised coordinates $\mathbf{q}$ is

$$\frac{\mathbf{Q}}{\delta Y} = \left(s^2\mathbf{A} - s\mathbf{D} - \mathbf{C}\right)^{-1} s^2\mathbf{B}, \tag{1}$$

where $\mathbf{A}$, $\mathbf{B}$, $\mathbf{C}$, and $\mathbf{D}$ are functions of the link masses m_i, link lengths L_i, equilibrium orientations α_{i0}, joint stiffnesses k_i, and joint damping c_i. The resonance frequencies of the undamped system correspond to values of $s = j\omega$ at which the denominator determinant vanishes:

$$\det\left(s^2\mathbf{A} - \mathbf{C}\right) = 0. \tag{2}$$

The damping matrix $\mathbf{D}$ is omitted because damping does not affect the resonance frequencies of lightly damped systems. For given values of m_i, L_i, and α_{i0}, this determinant is a function of s, k_1, k_2, and k_3. Evaluating (2) at the three experimentally measured resonance frequencies f_1, f_2, and f_3 (with $s_n = j\,2\pi f_n$) yields three equations in three unknowns:

$$\det\left(s_n^2\mathbf{A} - \mathbf{C}\right) = 0, \quad n = 1, 2, 3. \tag{3}$$

This system is solved symbolically. Six solution sets are obtained, but not all are physically plausible. There are a few limitations on further sorting physically plausible (k1 k2 k3). 1. The values must be positive real numbers. 2. The values should be at relatively similar level (e.g., one should not be 10 times larger than other), as they are from a continuous piece of plant tissue. In this study we set joint damping to a very small value, to have a clearer observation of resonances and anti-resonances. With the geometry and mass given in Table II, all possible values for (k1 k2 k3).

k1	k2	k3
$0.0037 + 0.0000i$	$0.0017 + 0.0000i$	$2.7931\text{e}{-05} + 0.0000\text{e}{+00}i$
$0.0301 - 0.0001i$	$1.3356\text{e}{-04} + 3.7408\text{e}{-05}i$	$4.0775\text{e}{-05} - 1.1258\text{e}{-05}i$
$0.0301 + 0.0001i$	$1.3356\text{e}{-04} - 3.7408\text{e}{-05}i$	$4.0775\text{e}{-05} + 1.1258\text{e}{-05}i$
$8.7275\text{e}{-04} + 0.0000\text{e}{+00}i$	$0.0016 + 0.0000i$	$1.2466\text{e}{-04} + 0.0000\text{e}{+00}i$
$9.1587\text{e}{-04} + 0.0000\text{e}{+00}i$	$3.2195\text{e}{-04} + 0.0000\text{e}{+00}i$	$5.9935\text{e}{-04} + 0.0000\text{e}{+00}i$
$0.0029 + 0.0000i$	$9.5911\text{e}{-05} + 0.0000\text{e}{+00}i$	$6.3970\text{e}{-04} + 0.0000\text{e}{+00}i$

TABLE III: Solution 2 and 3 are complex number, excluded. Solution 1 and 6 has k1 is more than 10x other joints, physically implausible, excluded. Solution 5 is identical to the manually found k in Table II. Solution 4 has the joint 2 to be stiffer than joint 1, which contradicts the thickness relation of the corresponding locations on the filament, we keep it for further investigation.

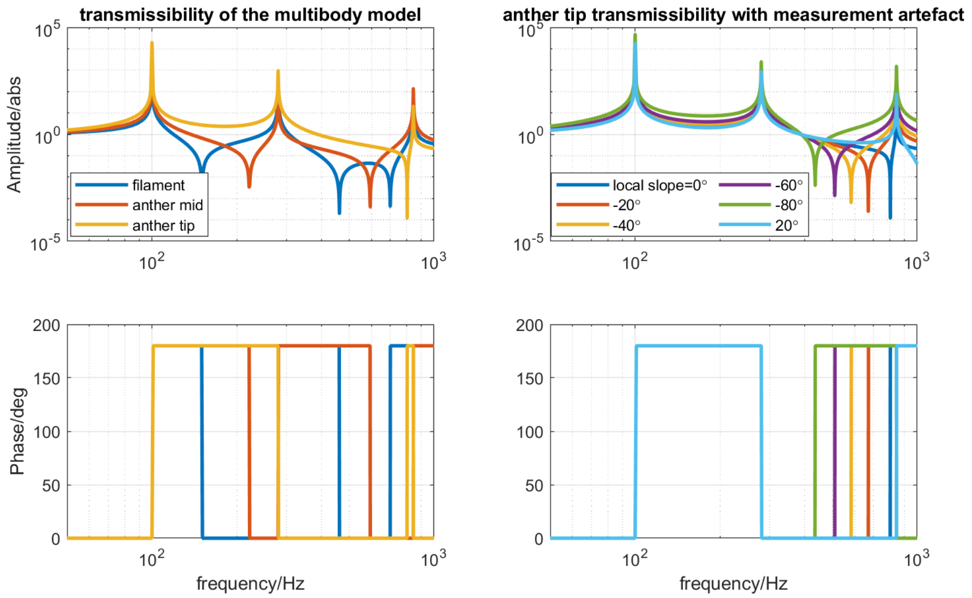

Fig. 22: The transmissibility without damping with stiffness value set 4. The left figures shows although all resonances match to the experimental results, the frequency of the anti-resonance in the anther tip is at a significantly higher frequency. After testing the effect of the measurement artefact with different local slopes, it can be concluded that this discrepancy cannot be explained by measurement artefacts.

Repeat this identifiability study for another set of link mass, while keeping the total mass unchanged. Use m1=2.2e-6;m2=1.5e-6;m3=0.9e-6, these values are based on the volume proportion of the filament, anther mid, anther tip segments in the FE model.

k1	k2	k3
$0.0031 + 0.0000i$	$0.0016 + 0.0000i$	$3.2780\text{e}{-05} + 0.0000\text{e}{+00}i$
$0.0269 - 0.0001i$	$1.2349\text{e}{-04} + 5.8921\text{e}{-05}i$	$4.0504\text{e}{-05} - 1.9085\text{e}{-05}i$
$0.0269 + 0.0001i$	$1.2349\text{e}{-04} - 5.8921\text{e}{-05}i$	$4.0504\text{e}{-05} + 1.9085\text{e}{-05}i$
$8.6844\text{e}{-04} + 0.0000\text{e}{+00}i$	$0.0015 + 0.0000i$	$1.2326\text{e}{-04} + 0.0000\text{e}{+00}i$
$9.0952\text{e}{-04} + 0.0000\text{e}{+00}i$	$3.0961\text{e}{-04} + 0.0000\text{e}{+00}i$	$5.8500\text{e}{-04} + 0.0000\text{e}{+00}i$
$0.0025 + 0.0000i$	$1.0497\text{e}{-04} + 0.0000\text{e}{+00}i$	$6.2404\text{e}{-04} + 0.0000\text{e}{+00}i$

TABLE IV: Similar to the previous case, set 1 and 6 are physically implausible, set 2 and 3 are complex. Set 4 has joint 2 as the stiffest joint, further investigate the resultant transmissibility.

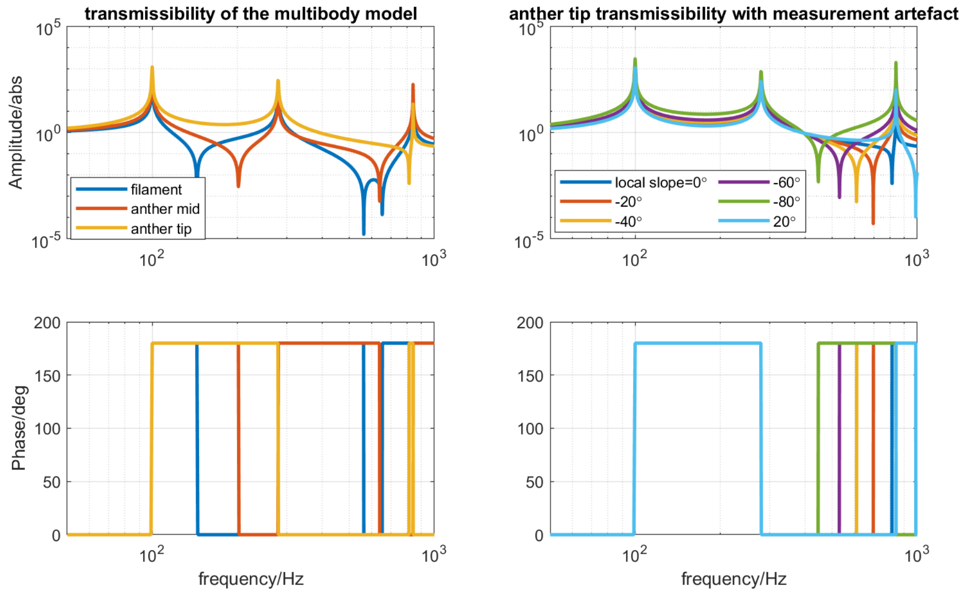

Fig. 23: Transmissibility with stiffness value set 4. The anti-resonance deviates significantly from experimental results and cannot be fixed by considering measurement artefact.

As shown, for a given set of link masses, when we know the frequencies of the three resonances of the individual specimen and its geometry, we can solve for joint stiffnesses of the multibody model. Although the initial solution is not unique, the uniqueness of solution can be obtained by screening the solutions with physical plausibility. Furthermore, when the link masses change from (1.8 2 0.8)*1e-6 to (2.2 1.5 0.9)*1e-6, the stiffnesses only change from (9.16 3.22 5.99)*1e-4 to (9.10 3.10 5.85)*1e-4, demonstrating that the uncertainty in link mass does not propagate into the stiffness. Plus, appendix A shows this model is not very sensitive to link mass. Therefore, uncertainty in link mass does not invalidate the capability of the multibody model of investigating sub-regional properties.

In this study, the link masses are assigned very roughly, because we were not able to weigh the sub-regional masses of the specimen. However, the second set of link masses proves that the stiffness solving method works as long as the link masses are determined and physically plausible, which includes sub-regional masses measured by cutting the specimen into the three segments and weigh each one on an analytical balance.

C. Cross-specimen geometry study of the multibody model

In appendix A, we show geometry alone does not determine the resonance frequencies of the stamen and sub-regional stiffness is the primary factor. Nevertheless, geometry still partly governs the qualitative topology of the transmissibility, including which dynamic features appear and in what order. To demonstrate, we use the multibody model to perform a cross-specimen study using three additional stamen specimen that span the full range of observed inter-specimen variation. The first additional specimen is an ”average” one, and the latter two specimens were selected because they differ markedly in one experimentally prominent dynamic feature: the frequency gap between the 2nd resonance and the anti-resonance at the anther tip.

For each specimen, the procedure is as follows.

1) The link geometry (lengths and equilibrium orientations) is extracted from the photograph of that specimen. The link masses and joint damping values are held fixed at the values in Table II.

2) Resonances frequencies are obtained from experimental data. For specimens whose 3rd resonance is not clearly identifiable from the high-quality transmissibility below 800 Hz, the 3rd resonance frequency is estimated from the raw measurement data between 800-1000 Hz, where the signal quality is reduced but the resonance peak remains visible.

3) Use the method in appendix B to solve the joint stiffnesses of the multibody model corresponding to this specimen. Note the anti-resonance at the anther tip is not part of the input.

4) The extracted stamen geometry, the solved joint stiffnesses, and the unchanged link masses and joint damping are used to generate the transmissibility of the multibody model. Another multibody model is constructed following previous steps, but with geometry in Table II, and the transmissibility is generated for comparison. The local slope at anther mid is set to 20°.

Below we present the photo of each specimen and the corresponding experimental and modelled transmissibility. All experimental results of filament and anther mid are drying effect fixed.

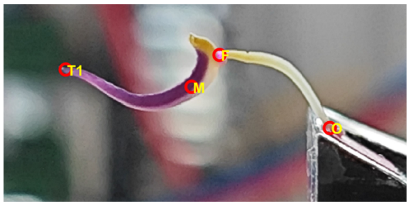

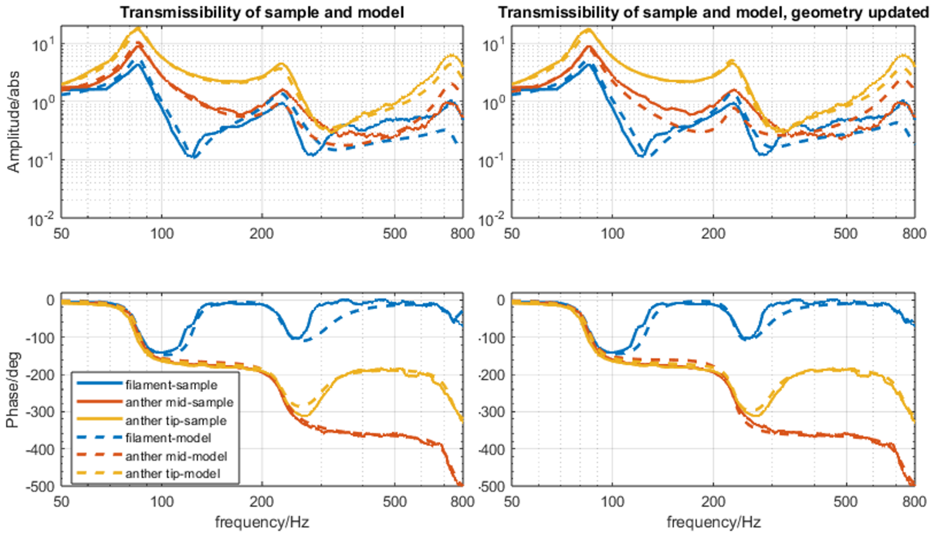

	Link 1	Link 2	Link 3
Length	19.8e-3	6.4e-3	19e-3
Orientation	147°	-132°	172°
Joint stiffness	6.09e-4	3.07e-4	3.90e-4

Fig. 24: A specimen with "average" geometry and dynamic features, the selected joints for geometry extraction, and solved joint stiffnesses for the geometry-updated multibody model (left). The measured transmissibility and the transmissibility of the model with geometry from Table II (middle). The measured transmissibility and the transmissibility with model geometry updated to the chosen specimen (right). Both models very well predicted the location of the anti-resonance at anther tip.

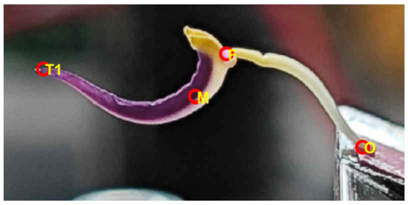

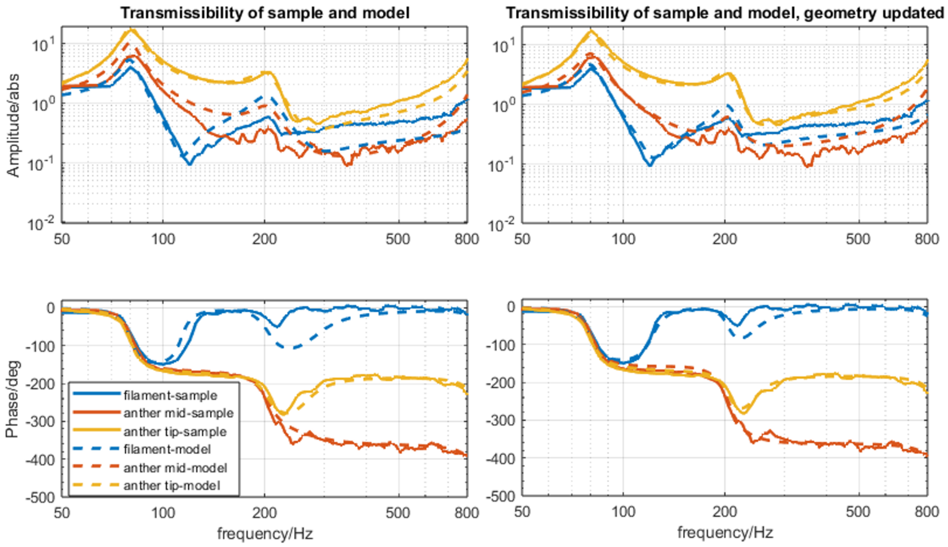

	Link 1	Link 2	Link 3
Length	19.8e-3	6.2e-3	18.6e-3
Orientation	146°	-126°	170°
Joint stiffness	5.10e-4	2.10e-4	6.11e-4

Fig. 25: A specimen with anther tip anti-resonance only 45Hz after the 2nd resonance(usually it is 70-150Hz), the geometry extraction and solved joint stiffnesses for the geometry-updated multibody model (left). Without updating the model geometry to the chosen specimen (middle), the anti-resonance at anther tip does not match experimental result, while updating the geometry improves this (right).

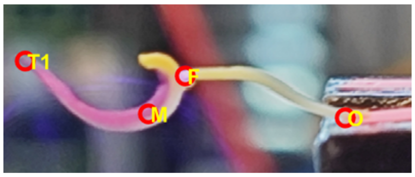

	Link 1	Link 2	Link 3
Length	19.8e-3	6.3e-3	16.1e-3
Orientation	165°	-134°	157°
Joint stiffness	5.63e-4	2.01e-4	6.74e-4

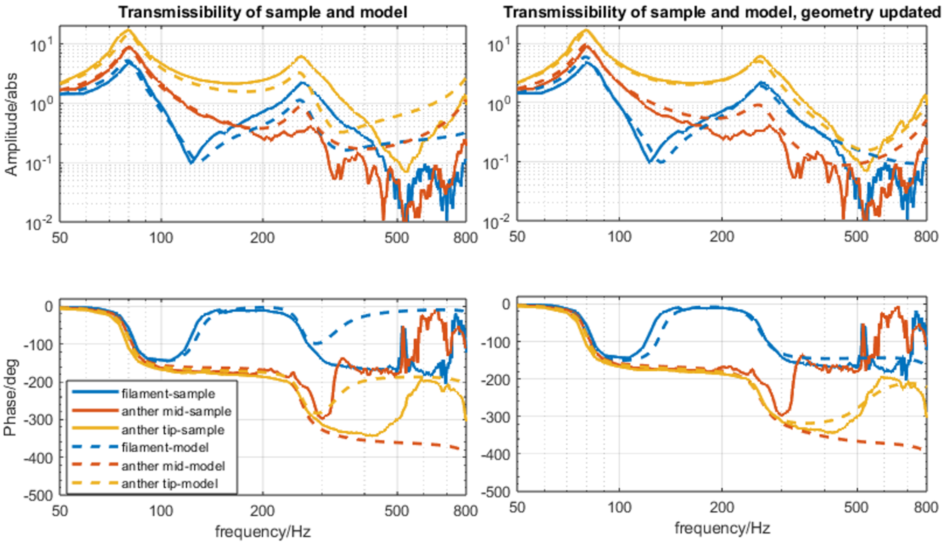

Fig. 26: A specimen with anther tip anti-resonance 260 Hz after the 2nd resonance and the solved joint stiffnesses for the geometry-updated multibody model (left). Without updating the model geometry to the chosen specimen (middle), the anti-resonance at anther tip does not match experimental result, while updating the geometry significantly improves the agreement (right).

Results above show that geometry plays an important role in shaping the overall structural dynamics of the stamen. If model geometry is not coherent to specimen, tuning sub-regional stiffnesses only matches resonance frequencies, while anti-resonances do not always match experimental results. This finding once again demonstrates that the rich dynamics of Medinilla *magnifica* stamens originates from its morphological complexity.

Several aspects of the transmissibility are not well reproduced with the multibody model. The response amplitudes deviate from the experimental data, because the joint damping values are carried over from the original value without re-tuning. The filament transmissibility shows visible discrepancy, primarily because the real filament is a continuous flexible body with infinite DoFs, whereas the model represents it with a single rigid link and one degree of freedom. In addition, the filament is measured last in the experimental sequence and therefore experiences the most drying, and the linear frequency interpolation used to compensate for drying may not fully capture the actual shift of all dynamic features. At the anther mid, most of the discrepancy may be attributed to the measurement artefact discussed in the main text. The correction method accounts for the artefact qualitatively, leaving residual deviation between the model and the experimental data.

In summary, with appendix A and appendix C, we not only discover the origin of dynamic properties of the stamen but also demonstrate the multibody model as a powerful tool for studying the effect of sub-regional properties and geometry on the structural dynamics. With the multibody model, modifying the geometry and other parameters are very direct and convenient and the computation is very fast. With the FE modelling, it is practically challenging to change the geometry without changing sub-regional masses and to change one sub-regional stiffness without affecting others. Furthermore, it will take much more time and effort to modify the model geometry and to execute the computation.
